# 3D Tissue-Engineered Vascular Drug Screening Platforms: Promise and Considerations

**DOI:** 10.3389/fcvm.2022.847554

**Published:** 2022-03-04

**Authors:** Isra Marei, Tala Abu Samaan, Maryam Ali Al-Quradaghi, Asmaa A. Farah, Shamin Hayat Mahmud, Hong Ding, Chris R. Triggle

**Affiliations:** ^1^Department of Pharmacology, Weill Cornell Medicine-Qatar, Doha, Qatar; ^2^National Heart and Lung Institute, Imperial College London, London, United Kingdom

**Keywords:** 3D drug screening, tissue engineered blood vessels, stem cells, scaffolds, self-organization and self-assembly, 3D bioprinting, bioreactors, personalization

## Abstract

Despite the efforts devoted to drug discovery and development, the number of new drug approvals have been decreasing. Specifically, cardiovascular developments have been showing amongst the lowest levels of approvals. In addition, concerns over the adverse effects of drugs to the cardiovascular system have been increasing and resulting in failure at the preclinical level as well as withdrawal of drugs post-marketing. Besides factors such as the increased cost of clinical trials and increases in the requirements and the complexity of the regulatory processes, there is also a gap between the currently existing pre-clinical screening methods and the clinical studies in humans. This gap is mainly caused by the lack of complexity in the currently used 2D cell culture-based screening systems, which do not accurately reflect human physiological conditions. Cell-based drug screening is widely accepted and extensively used and can provide an initial indication of the drugs' therapeutic efficacy and potential cytotoxicity. However, *in vitro* cell-based evaluation could in many instances provide contradictory findings to the *in vivo* testing in animal models and clinical trials. This drawback is related to the failure of these 2D cell culture systems to recapitulate the human physiological microenvironment in which the cells reside. In the body, cells reside within a complex physiological setting, where they interact with and respond to neighboring cells, extracellular matrix, mechanical stress, blood shear stress, and many other factors. These factors in sum affect the cellular response and the specific pathways that regulate variable vital functions such as proliferation, apoptosis, and differentiation. Although pre-clinical *in vivo* animal models provide this level of complexity, cross species differences can also cause contradictory results from that seen when the drug enters clinical trials. Thus, there is a need to better mimic human physiological conditions in pre-clinical studies to improve the efficiency of drug screening. A novel approach is to develop 3D tissue engineered miniaturized constructs *in vitro* that are based on human cells. In this review, we discuss the factors that should be considered to produce a successful vascular construct that is derived from human cells and is both reliable and reproducible.

## Introduction

The major breakthroughs in biological and chemical sciences are not equally translated into drug discovery and the consequent development of effective treatments (1). Despite the promise of drug developments in the pre-clinical phase, only 10% of the drugs that enter phase 1 clinical trials are expected to advance to FDA approval (2, 3). Besides factors such as the increased cost of clinical trials and sponsors complex regulatory processes, there is also a gap between the currently existing pre-clinical screening models and the *in vivo* clinical studies on humans (2). This gap is mainly caused by the lack of complexity in the currently used 2D cell culture-based screening systems, which do not accurately reflect human physiological responses (1, 4, 5). Thus, more reliable and accurate drug screening systems are required to bridge the gap between the *in vitro* pre-clinical screening platforms and human clinical trials.

During drug development, *in vitro* screening using high-quality tools is an important step prior to translating drug use to the clinic (6). Cell-based drug screening is a widely accepted model that is used prior to the more complex animal tests and clinical trials (7). These screening methods provide initial indication of the therapeutic efficacy of the drug and potential cytotoxicity (6). However, *in vitro* cell-based evaluation could in many instances provide contradictory findings to the *in vivo* testing in animal models and clinical trials (1). This drawback is related to the failure of these 2D cell culture systems to recapitulate the human physiological environment in which these cells reside (8). Vascular cells in their native environment reside within a complex physiological setting, where they interact with and respond to neighboring cells, extracellular matrix, mechanical stress, blood shear stress, and many other factors. These factors in sum affect cellular responses and the specific pathways that regulate variable functions such as proliferation, apoptosis and differentiation (1). Although pre-clinical *in vivo* animal models provide this level of complexity, cross species differences might also result in contradictory results when compared to clinical trials (9). Thus, there is an urgent need to mimic the human physiological conditions in pre-clinical studies to improve drug screening platforms (1). A novel approach is to use patients' stem cells to develop 3D tissue engineered constructs *in vitro*. Such platform will also offer the potential for personalized drug testing to accommodate to patients' specific needs (10). Such an approach is already in use to treat cancer patients (11).

**Figure 1:**
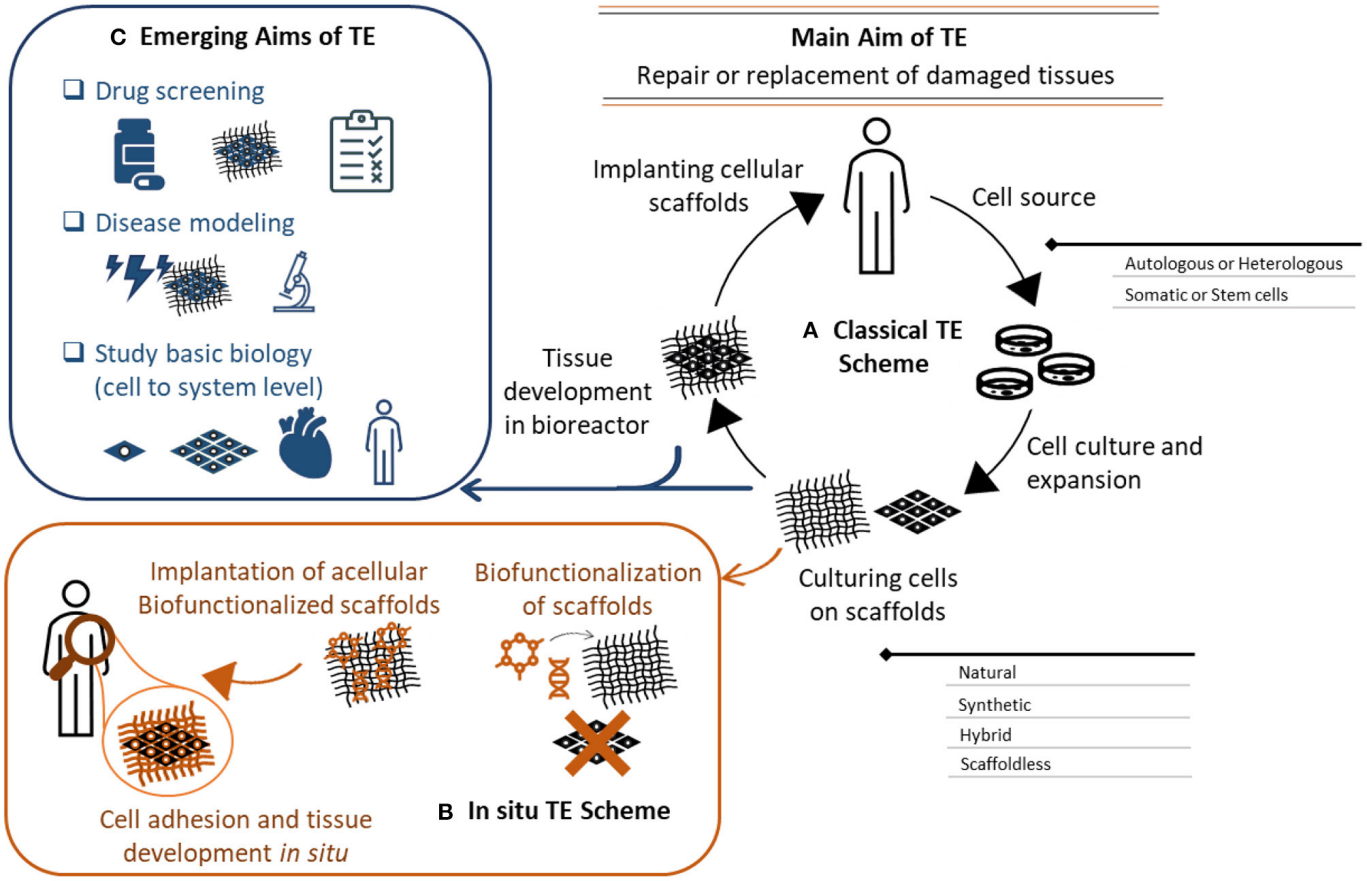
Tissue engineering aims, concept, and evolution. **(A)** Classical *in vitro* tissue engineering (TE) relys on the use of a cell source (autologous or heterologous), cultured into a 3D scaffold. The cellular scaffold is then incubated in a bioreactor to influence cells growth, extracellular matrix secretion and subsequent tissue formation. The developed tissue is then used to replace damaged tissues. This scheme has evolved to an *in-situ* TE scheme **(B)**, where acellular scaffolds are biofunctionalized with bioactive molecules to instruct cell adhesion and tissue formation. These instructive scaffolds are implanted to induce tissue formation *in situ*. **(C)** The applications of *in vitro* tissue engineering also extend to drug screening, disease modeling, and cell/system biology studies. The complexity of these constructs provides a more biomimetic platform that can recapitulate the *in vivo* physiology of the human body. These systems also provide a better understanding of cell biology and functions from cells to systems level.

Tissue engineering is a multi-disciplinary field combining engineering, materials and biological sciences with the aim to repair or replace damaged tissues. As first described by Langer and Vacanti in 1993, the initial concept of tissue engineering was to grow autologous cells supported with a scaffolding material under controlled conditions *in vitro* to develop a viable construct (12) (Figure 1A). This concept has since evolved, and the reconstruction of tissue engineered viable substitutes has now been extensively investigated with and without the support of 3D scaffolds (13) (Figure 1B). Tissue engineering offers a promising solution to treat a variety of diseases, including those affecting the cardiovascular system (14). The application of tissue engineering is not only restricted to therapeutics and regenerative medicine, but it also extends to pre-clinical studies, drug discovery, and disease modeling (Figure 1C). This is due to the level of complexity offered by tissue engineering constructs, and the ability to mimic the microvascular physiological environment of native cells (5). Several studies investigated the use of 3D vascular grafts and tissue-engineered conduits for the development of a drug-screening platform. Despite their promise, careful selection and evaluation of these methods is required prior to their implementation. We discuss here the factors that should be considered to produce a successful construct that is both reliable and reproducible.

## Considerations for the Development of 3D Vascular Grafts for Drug Screening

**Figure 2:**
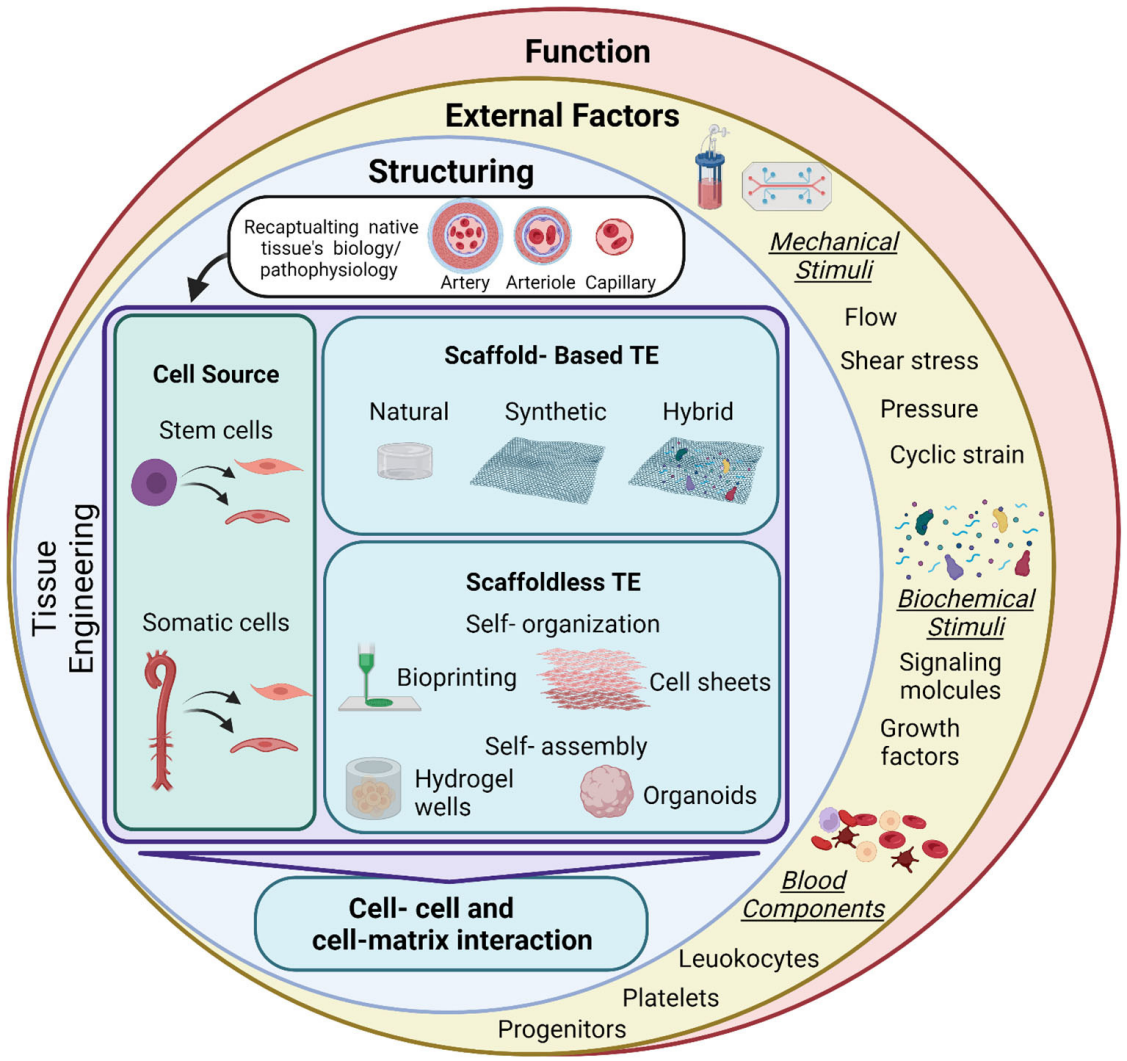
Summary of tissue engineering approaches and applications for drug testing. Tissue engineering (TE) is used to develop vascular grafts of different sizes and structures to recapitulate the native biology/pathophysiology of the target tissue. Investigated cell sources are of somatic or stem cell origin. Vascular grafts are developed through either scaffold-based or scaffoldless TE approaches. Scaffold based approaches adopt the classical TE scheme and rely on the culture of cells on natural, synthetic or hybrid scaffolds to provide structural support and stability of the construct. Emerging scaffoldless techniques rely on self-organization or self-assembly of the cells and their ability to secrete extracellular matrix to develop the vascular tissue. Self-organization is achieved through bioprinting or cell sheet multi-layering techniques. Self-assembly is influenced in hydrogel wells to develop self-organized 3D tissues derived by the differential adhesion hypothesis. These TE techniques lead to the development of a 3D construct, characterized by cell-cell and cell-matrix interactions. This provides a better model for cells than the conventional 2D monolayer cultures that are currently used for drug screening. Exposing these constructs to external factors will increase the complexity of the system and provide a more accurate biomimetic substrate for drug testing. These factors include mechanical stimuli (fluid flow/shear, pressure, and cyclic strain), biochemical stimuli (signaling molecules and growth factors) and blood components (leukocytes, platelets, and progenitor cells). These factors will influence the function of the construct. Mimicking pathological conditions could also aid in modeling disease conditions, which will provide a more accurate representation for drug testing for specific pathologies. Figure was created by BioRender.com.

The development of 3D tissue engineered blood vessels dates back to 1986, when Weinberg and Bell reported the construction of a multi-layered blood vessel formed of endothelial cells, smooth muscle cells and collagen on a Dacron mesh (15). This early model demonstrated the basic requirements that should be matched by a tissue-engineered vessel to mimic their native counterparts. This has been followed by many attempts to improve these tissue-engineered systems and have shown success both *in vitro and in vivo*. Utilizing these advances to improve the available drug screening and discovery platforms has also been a target. To that end, the tissue engineered construct should mimic the physiological structure and function of their counterparts, and for that the structure, functions, and surrounding environment should be matched to the physiological setting. Here we summarize the different approaches that can be used to develop 3D blood vessels for drug screening purposes, the cell sources and scaffolds to formulate a 3D construct, and considerations to achieve the native structure and function. We also highlight the importance of the physiological dynamic conditions needed to influence structure and function (Figure 2).

### Approach

**Figure 3:**
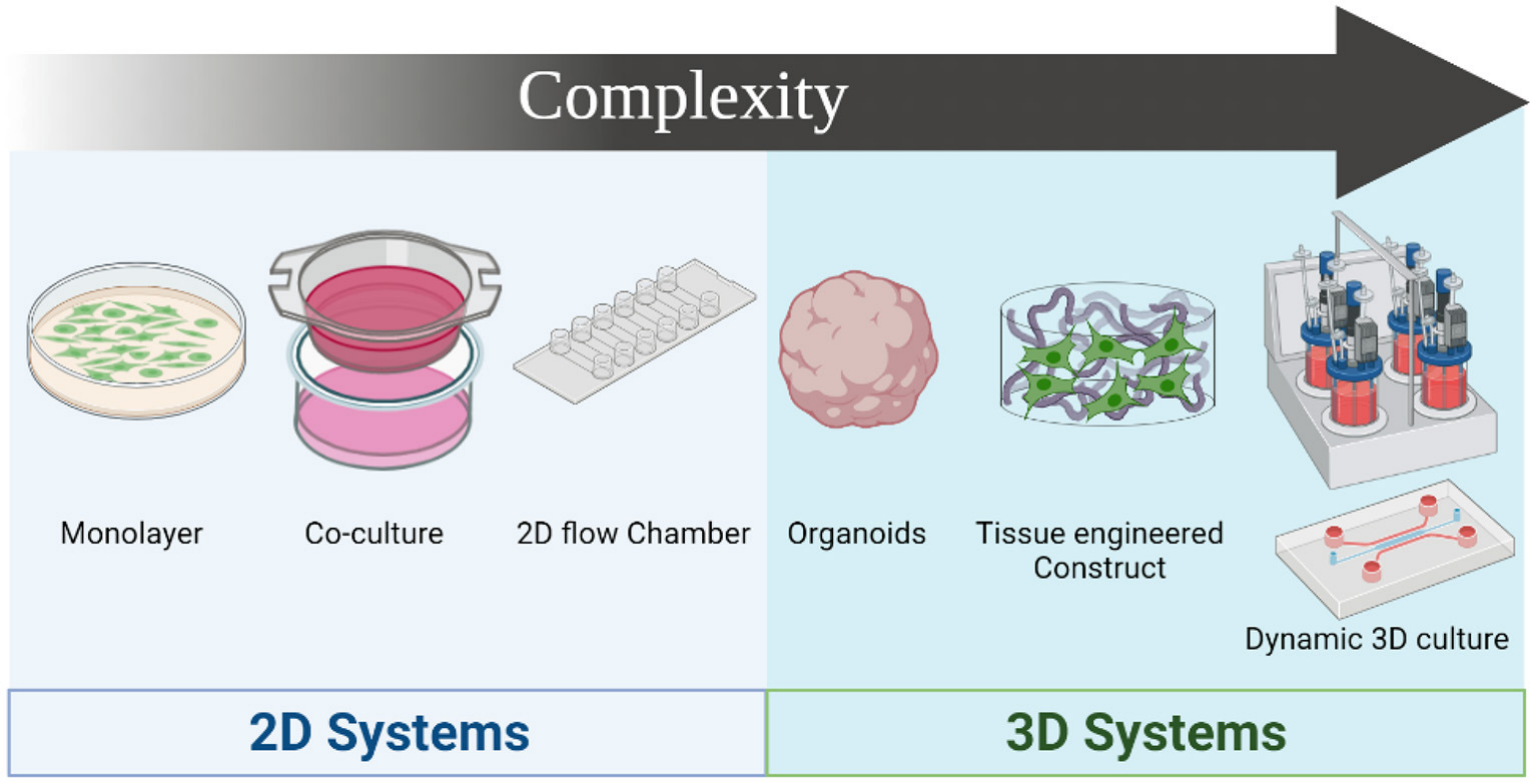
Drug screening systems evolution based on complexity. The simplest model is the culture of single cell monolayers on 2D format, followed by the coculture of 2 different cell types in 2D format, and the use of 2D flow systems. 3D culture systems provide higher level of complexity, starting with organoids that provide 3D structures with histological similarities to native tissues. Development of 3D constructs using traditional tissue engineering approaches and 3D bioprinting provides higher precision in mimicking the microstructure of the native tissue, and combining these constructs with dynamic systems such as bioreactors or utilizing organ-on-a-chip in combination with microphysiological flow recapitulates the physiological setting of the blood vessel. Figure was created by BioRender.com.

3D tissue engineered vascular grafts have been investigated for drug screening and disease modeling using “traditional” tissue engineered constructs, bioprinted grafts, organ-on-a-chip models, and organoids (Table 1). Each of these approaches provides a level of complexity and organization to recapitulate the physiological setting (Figure 3). Traditional tissue engineered constructs for example, have been developed using variable methods to achieve cell organization, matrix production and structural integrity. These include scaffold-supported and scaffoldless constructs based on a variety of cell types, and in combination with flow inducing systems as will be covered in detail in the next sections. Examples of the use of these constructs in drug screening are listed in Table 2. One recent example is the development of tissue engineered blood vessels through layer-by-layer assembly of a medial layer composed of human coronary artery smooth muscle cells in type I collagen, covered with an intimal layer composed of a human umbilical vein endothelial cell (HUVEC)-seeded aligned PLA nanofibers scaffold (17). The model was used to perfuse human blood or platelets under physiological flow conditions using a parallel-plate flow chamber. This allowed the testing of intact and damaged vessels and provided an antithrombotic drug testing platform mimicking the *in vivo* thrombosis models based on the use of ketamine (17).

**Table 1:** Approaches for the development of 3D drug screening systems, their advantages, and limitations (16).

**Classical tissue engineering**		**3D bioprinting**	
Scaffold-supported and scaffoldless constructs seeded with cells, and incubated in a bioreactor	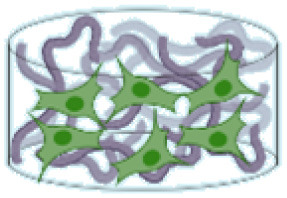	Layer-by-layer deposition of a bioink composed of cells, growth factors and biomolecules; with or without biomaterials to develop the target tissue or organ	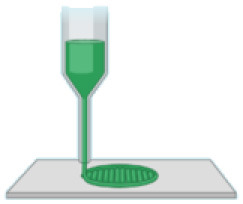
**Advantages:** Co-culture model Adjustable to microplates Reproducible Applicable for high throughput screening		**Advantages:** Co-culture model Precision Tailored micropatterns/architecture High throughput production Miniaturized models	
**Limitations** Variability Requires standardized methods and materials		**Limitations** Requires optimizations of cells and biomaterials Limitations of bioinks and bioprinters Cell viability	
**Organ on a chip**		**Organoids**	
3D microfluidic systems that combine the use of human cells and microphysiological flow to mimic the physiological environment.	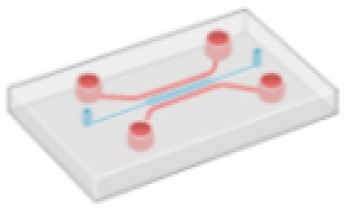	The culture of stem cells, and their differentiation and self-organization to simplified 3D structures with histological similarity to native tissues	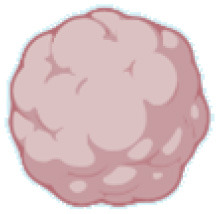
**Advantages:** Co-culture model Tailored microenvironment/architecture Microfluidics		**Advantages:** Histological similarity to native tissues Simplified and miniaturized 3D representation of body organs	
**Limitations** Difficult to apply to high throughput screening		**Limitations** Variability Maturity level	

**Table 2:** Examples of 3D vascular drug screening models developed using different tissue engineering approaches including traditional tissue engineering, self-organization (3D bioprinting, cell sheets, and organoids), and self- assembly.

**Approach**	**Cell type (s)**	**Scaffold/biomaterial**	**3D model**	**References**
Traditional tissue engineering	Human coronary artery smooth muscle cells and HUVECs	Type I collagen, and aligned PLA nanofibers scaffold	**Model** (17, 18)**:** Layer-by-layer assembly of a medial layer composed of human coronary artery smooth muscle cells in type I collagen, covered with an intimal layer composed of a HUVEC-seeded aligned PLA nanofibers scaffold. **Application** (17)**:** Thrombosis model: Perfusion of human blood or platelets under physiological flow conditions using a parallel-plate flow chamber. **Application** (18)**:** Real time monitoring of cytosolic Ca^2+^ in human platelets exposed to tissue engineered vessels to quantitatively compare the construct ability to promote or prevent platelet activation.	(17, 18)
	Endothelial and smooth muscle cells derived from human embryonic stem cells and iPSCs	Fibrin gels	**Model:** Cells were cultured into fibrin gels to induce 3D tissue formation. **Application**: The system was used to test a high-throughput screening strategy to assess chemical toxicity and drug efficacy.	(19)
	Human neonatal dermal fibroblasts or human bone marrow-derived MSCs	Rat-tail collagen I matrices	**Model:** Dense collagen gel matrices were developed by embedding human neonatal dermal fibroblasts or human bone marrow derived MSCs in rat-tail collagen I. The seeded matrix was then poured into a mandrel and allowed to gel. **Application:** Custom-made perfusion bioreactor chamber to test pharmacological and immunological responses of tissue engineered vascular grafts.	(20)
	Primary or iPSC-derived smooth muscle cells and EPCs	Collagen gel	**Model:** Medial cells (primary or iPSC-derived smooth muscle cells) embedded in a mixture of collagen gel and injected into molds to fabricate arteriole-scale human vessel grafts that are then endothelialized in the perfusion chamber.	(21)
	Vascular cells generated from PBMCs-derived iPSCs	PGA-P4HB starter matrices	**Model:** Vascular cells were used to seed tubular non-woven synthetic scaffolds and formulate small diameter vascular grafts under static and pulsatile flow conditions **Application:** Autologous PBMC derived iPSC-derived vascular constructs could be used for disease modeling and drug testing.	(22)
Self-organization (3D bioprinting, cell sheets, organoids)	Smooth muscle and endothelial cells derived from human PSCs	Fibrin matrix	**Model:** Induced self-organization of smooth muscle and endothelial cells derived from human PSCs in fibrin matrix using vascular endothelial growth factor to form microvasculature constructs. **Application:** 3D constructs arrayed in high throughput were used to screen a library of environmental and clinical vascular toxicants for immunological and toxicological responses.	(19)
	Human smooth muscle cells derived from pulmonary hypertension patients	–	**Model:** Culture of the media layer of blood vessel stimulating the thickening of a 3D media layer formed of human smooth muscle cells derived from pulmonary hypertension patients. **Application:** Effect of pulmonary hypertension drugs to suppress medial thickening.	(23)
	Human MSCs and EPCs	–	**Model:** Scaffoldless aligned human MSC sheets coated with human EPCs and cultured in a rotating wall bioreactor. **Application:** Tested the vascoactivity of the developed human cell-based endothelialized grafts in response to phenylephrine. This microphysiological system could be used for autologous drug screening.	(8)
	PSCs differentiated into endothelial cells and pericytes (24, 25) HUVECs, and smooth muscle cells derived from human ESCs and human iPSCs (26)	Matrigel/ collagen Methylcellulose-based hydrogel system (26)	**Model** (24, 25)**:** Organoids model of diabetic vasculopathy. **Model** (26)**:** Organoid co-culture model of smooth muscle and endothelial cells. Determined vascularization of organoids embedded in collagen/fibrinogen/fibronectin hydrogel. **Application** (26)**:** *in vitro* co-culture model to study paracrine interactions between vascular cells. The system mimics physiological assembly of vessels and could be used for drug development and preclinical metabolic and toxicology studies.	(24, 25) (26)
	Endothelial cells	Polylactic acid for fused-filament 3D fabrication and PDMS for the cast	**Model:** 3D printing/microfluidics model of *in vivo* blood vessel network biology from healthy and diseased tissues. 3D printing of blood vessel images using fused-filament 3D fabrication by Polylactic acid. The 3D printout is cast in PDMS and dissolved, to produce the channels which are then lined with endothelial cells. **Application:** This model could be an effective tool to study drugs interactions with the endothelium under physiological flow conditions.	(27)
	Endothelial and smooth muscle cells	Nanoengineered hydrogel-based cell-laden bioinks	**Model:** 3D bioprinting of anatomically accurate, multi-cellular blood vessels using Nanoengineered hydrogel-based cell-laden bioinks. **Application:** Upon cytokine stimulation and blood perfusion, this 3D bioprinted vessel is able to recapitulate thromboinflammatory responses.	(28)
	HUVECs and MSCs	Gelatin-norbornene hydrogel cast	High throughput sample-agnostic bioreactor system, that was tested on vascular grafts made of HUVECs and MSCs encapsulated in gelatin-norbornene hydrogel cast into stereolithography 3D bioprinted well inserts.	(29)
Self-assembly	Smooth muscle cells	Pre-structured annular agarose well	**Model:** Smooth muscle cells were seeded into a pre-structured annular agarose well, which induced cell aggregation and self-assembly to develop tissue rings. **Application:** use the developed rings to formulate tissue tubes based on ring fusion, in presence of gelatin microspheres (30) that can deliver growth factors and influence cell phenotype (31).	(30, 31)
	Smooth muscle cells derived from human iPSCs	Agarose well systems	Development of vascular rings in agarose well systems using highly enriched functional smooth muscle cells derived from human induced pluripotent stem cells	(32)
	HUVECs and aortic smooth muscle cells	Agarose well systems	The use of agarose well systems in combination with cellularized microcarriers composed of gelatin microcarriers loaded with HUVECs and aortic smooth muscle cells to develop tubular structures.	(33)

Bioprinting is another approach that allows the specific organization of cells into structures that mimic the natural tissue. The approach is similar to traditional 3D printing, where a bioink composed of cells, growth factors and biomolecules; with or without biomaterials; is deposited in a layer-by-layer manner to develop the target tissue or organ (34). The use of bioprinting overcomes some of the limitations of traditional tissue engineering techniques, as it provides greater level of precision when depositing cells/biomaterials, which influences their arrangement and spatial interaction (35, 36). The process is automated allowing for controlled micropatterning of cells and extracellular matrix (ECM) in a bottom-up approach (36). Bioprinting modalities could be categorized to inkjet/droplet, microvalve, extrusion-based, laser-assisted, and stereolithography bioprinting. Since the requirements and mechanics of bioprinting are outside the scope of this review, we refer the reader to some reviews that provide an overview on bioprinting (37–40). Here, we provide a brief description of these modalities, and throughout the review, we discuss some of their applications in 3D vascular drug testing. These modalities differ in the mode and speed of deposition, spatial resolution, and cell density/viability. Inkjet bioprinting is achieved by the controlled depositing of droplets into the substrate. In microvalve printing, the deposition of droplets is generated by the opening/closing of a microvalve controlled by pneumatic pressure. Extrusion-based printing depends on the continuous extrusion of bioink filaments through a nozzle in a controlled manner. Laser-assisted bioprinting uses a pulsed laser beam as an energy source to guide the deposition of the bioink into a substrate (41). Stereolithography bioprinting uses an ultraviolet light to cure layers of photosensitive polymer in stacks that form the 3D structure (42). Multi-material bioprinting techniques has also been introduced to improve the formation of complex multi-component structures (43). These systems include multiple print heads or nozzles that allow the bioprinting of different materials and cells. These systems include multi-nozzle, coaxial, and microfluidics-assisted bioprinters. We refer the read to a comprehensive review on this topic (43). Bioinks are key components of 3D bioprinting, and their selection depends on the target tissue, the cell type and the bioprinting technique (40, 44). Bioinks could be composed of natural or synthetic materials such as alginate, gelatin, collagen, hyaluronic acid, Matrigel, polycaprolactone, polyethylene glycol, and polyvinylpyrrolidone (44). Additionally, bioinks made of decellularized ECM have been developed (44). Cell pellets and aggregates could also be used to fabricate scaffold-free constructs (44). Bioinks can be functionalized with bioactive molecules to provide controlled microenvironment which promotes the formation of a more biomimetic construct (45). Examples of the applications of 3D bioprinting for drug testing are summarized in Table 2.

Organ-on-a-chip systems are miniaturized systems that combine tissue engineering and microphysiological flow, allowing drug testing on organ micro-models developed using human cells, thus marinating human genetic background under complex physiological settings. Some examples have been used to study pulmonary hypertension (46), diabetes (47), and thrombosis (48). We refer the reader to some recent comprehensive reviews that cover the use of these systems for vascular drug screening (49–51).

Culturing organoids is another approach that provides a simplified and miniaturized 3D representation of body organs. This system relies on the culture of stem cells (52), and their differentiation and self-organization to provide organoids with histological similarity to native tissues. The development of human blood vessel organoids has been reported using pluripotent stem cells differentiated into endothelial cells and pericytes (24, 25). Wimmer et al. reported the development of these organoids and their use to model diabetic vasculopathy (24).

### Cell Source

One of the main factors that dictate the success of vascular grafts is the cell source. The cell sources for blood vessel tissue engineering applications has been reviewed intensively (53–57). Here we summarize some of the potential sources that could be used specifically to develop 3D vascular grafts for drug screening (Table 3). The sources of cells used for tissue engineering have either a somatic or stem cell origin. Choice of cells should be made relying on their ability to differentiate to cells from the vascular lineage (i.e., smooth muscle cells, endothelial cells and pericytes) to recapitulate the structure of the native blood vessel. The ideal cell source that will allow a more personalized approach for drug testing and screening would be an autologous source. Patient-derived autologous somatic cells isolated directly from native tissues are ideal in reflecting the *in vivo* phenotype and function of the tissue. However, these cells require invasive methods to isolate and have limited replication capacity (56, 69).

**Table 3:** Summary of the cell sources for vascular tissue engineering, their advantages, and limitations.

**Cell type**	**Source**	**Differentiation to vascular cells**	**Advantages**	**Limitations**	**Examples**
Somatic cells	Somatic tissues	Fully differentiated at the time of isolation	• Standardized isolation methods • Reflect the phenotype of native vascular cells	• Invasive isolation methods • Limited replication capacity	(33)
Induced pluripotent stem cells	Skin- derived, EPCs-derived	Differentiate into vascular endothelium and smooth muscle cells (58)	Robust source of autologous cells	• Low reprogramming efficiency • Need to establish more robust differentiation protocols. • Genetic and epigenetic alterations	(19, 22, 32, 59, 60)
Mesenchymal stem cells	Bone marrow, Cord and peripheral Blood	Differentiate into vascular endothelial and smooth muscle cells (61)	• Could be isolated from a wide range of tissues • Antithrombotic properties	• Difficult isolation and identification • Heterogeneity of MSC population • Lower regenerative potential in some pathologies such as diabetes	(8, 20, 31)
Adipose derived stem cells	Adipose tissue (stromal vascular fraction)	Differentiate to smooth muscle and endothelial cells	Similar to MSCs in terms of morphology, phenotype and differentiation potential	• Differentiation to fully mature endothelial phenotype is limited • Altered cytoskeletal integrity in ASCs engineered tissues (62)	(63)
Endothelial progenitor cells	Cord and peripheral Blood, bone marrow	Differentiate to mature endothelial cells, with potential of endothelial-mesenchymal transition	• Accessible cell source • Stable mature endothelial phenotype (late EPCs) • Robust proliferation	• Relatively prolonged and expensive isolation methods • Heterogeneity and uncertainty of the resulting phenotypes from different origins/isolation methods • Cells emergence could be lower in certain pathologies (e.g., Diabetes and cardiovascular disease)	(64–67)
Embryonic stem cells	Early-stage embryos (inner cell mass of a blastocyst)	Differentiate to smooth muscle and endothelial cells	Could be maintained for long durations in culture	• Ethical, political and religious controversies • Sourcing difficulties • Low efficiency to generate stable endothelial cell phenotype.	(68)

The adult tissues also contain small populations of undifferentiated, but committed, non-embryonic stem cells known as adult or somatic stem cells. These cells have the ability to self-renew and differentiate to mature cells (70), however, their differentiation is only limited to specialized cell types from the same germ origin, or in a limited fashion to other cell lineages, thus they are considered as uni- or multi-potent stem cells (71). Theses stem cells are found in most adult tissues, such as bone marrow, blood vessels, heart tissues and valves, adipose tissue, muscle, and skin (72). Recent studies demonstrated the adaptability of somatic stem cells under specific stimuli or injury and their ability to perform distinct functions (72). Furthermore, the reprogramming of somatic stem cells to induced pluripotent stem cells demonstrates a breakthrough in disease modeling and drug screening (71). Although somatic stem cells have limited differentiation capacity, they represent a potential resource of cells capable of differentiation and can be used for drug screening. Here, we briefly discuss the use of induced pluripotent stem cells, mesenchymal stem cells, adipose derived stem cells, and endothelial progenitor cells, in addition to the pluripotent cell source: embryonic stem cells.

Induced pluripotent stem cells (iPSCs) are stem cells generated from somatic cells that have been reprogramed to resemble embryonic stem cells (58). These stem cells have the ability to differentiate into a wide range of specified cells from all three germ layers, such as cardiomyocytes, neurons, and hepatocytes (73). Additionally, iPSCs have shown the ability to differentiate into vascular endothelium and smooth muscle cells (58). There are three main iPSCs differentiation strategies, including embryoid bodies, co-culture with stromal cells, and ECM guided differentiation. Each strategy has its own advantages and limitations (56). Human iPSCs were successfully differentiated into vascular cells *in vitro*, and differentiation strategies have been reviewed for endothelial (58, 74) and smooth muscle cells (75, 76). Furthermore, the use of iPSCs-derived vascular cells for 3D drug screening was investigated in several studies (19, 22, 59). For example, Titmarsh et al. successfully derived endothelial and smooth muscle cells from human embryonic stem cells and iPSCs, confirmed their expression and functional characteristics, and cultured these cells into fibrin gels to induce 3D tissue formation. The system was used to test a high-throughput screening strategy to assess chemical toxicity and drug efficacy (19). Generali et al. used peripheral blood mononuclear cells (PBMCs)-derived iPSCs to generate vascular cells, which were then used to seed tubular non-woven synthetic scaffolds and formulate small diameter vascular grafts (22). Another study by Nakayama et al. developed grafts based on primary or iPSCs-derived vascular cells seeded on aligned nanofibrillar collagen scaffolds (60). Other applications of iPSCs to assess drug-induced vascular toxicity were recently reviewed by Tu et al. (59).

Mesenchymal stem cells (MSCs) are another cell source that is widely studied for their potential in tissue engineering and regenerative medicine. MSCs can be isolated from different tissues including bone marrow, umbilical cord, adipose tissue, and peripheral and cord blood (57). These stem cells can also be differentiated from iPSCs (77). MSCs have the ability to self-renew and differentiate into different cell linages (61). According to the International Society for Cellular Therapy (ISCT), MSCs are defined by their multi-potent differentiation potential, adhesion to plastic surfaces, and characteristic cell antigen expression (78). MSCs express markers including CD105, CD73, and CD90, and lack the expression of hematopoietic markers amongst others (CD45, CD34, CD14 or CD11b, CD79a or CD19, and HLA class II) (78). Differentiation of MSCs to vascular cell types could be confirmed using cell specific markers (α-smooth muscle actin, SM22, smooth muscle myosin, and calponin for smooth muscle cells, and CD31, VE cadherin and von Wilborn factor for endothelial cells), and functions (contraction and tube formation, respectively) (61). The differentiation of MSCs to smooth muscle cells is promoted in presence of transforming growth factor-1 (TGF-β1) (79) and contractility is enhanced. Kinnaird et al. also suggest that MSCs have paracrine activity, as they secrete cytokines and growth factors such as platelet-derived growth factor-B receptor, fibroblast growth factor-2 and hepatocyte growth factor, which promote their differentiation (80). Additionally, MSC conditioned media was shown to enhance the proliferation and migration of endothelial and smooth muscle cells (80). Many of the drug testing models described in the literature rely on the use of MSCs for the development of vascular grafts. For example Jung et al. have developed a tissue engineered blood vessel from aligned human mesenchymal cell sheets coated with human endothelial cells (8). Fernandez et al. have also generated tissue engineered blood vessels made of human neonatal dermal fibroblasts or human bone marrow-derived mesenchymal stem cells supported by an extracellular matrix scaffold made of collagen gel and endothelialized using blood endothelial progenitor cells (20).

Another source of multi-potent stem cells is adipose derived mesenchymal stem cells (ASCs). ASCs are isolated from the stromal vascular fraction obtained after the centrifugation of adipose tissue harvested from liposuction (81). ASCs are similar to bone marrow derived MSCs in terms of their morphology, phenotype, and differentiation potential (82). ASCs have the potential to differentiate into all mesenchymal cell linages to give rise to adipogenic, osteogenic, chondrogenic, and myogenic cells. Additionally, ASCs were shown to differentiate to smooth muscle and endothelial cell phenotypes. Differentiation to smooth muscle cells has been described upon stimulation with factors such as TGF-β1 (55, 83), bone morphogenetic protein 4 (55), Angiotensin II (84), and platelet-derived growth factor-BB (83). Differentiation to endothelial cells was reported to be induced by methylcellulose semi solid media (85), growth factors [such as endothelial cell growth supplement derived from bovine hypothalamus (86), and VEGF (87)], and shear stress (86, 87). Differentiation to endothelial cells was also shown to be successful form elderly patients with cardiovascular diseases (88). However, the differentiation potential of ASCs to a fully mature endothelial phenotype is thought to be limited as was indicated by the hypermethylation of the endothelial-specific promoters CD31 and CD144, even following endothelial stimulation (89). It was suggested that hypermethylation of lineage-specific promotors might repress cell differentiation potential to these lineages, while hypomethylation is potentially permissive, with no predictive value on the differentiation potential (90). Thus, the methylation state of these endothelial-specific promotors in ASCs indicate their limited differentiation ability to endothelial cells (89, 90). Despite that, several reports described the development of vascular grafts based on ASCs for *in vitro* and *in vivo* applications. For example, Zhou et al. used human ASCs to develop a bilayered small diameter blood vessel (63). In this study, ASCs-derived smooth muscle cells were seeded into electrospun polycaprolactone-gelatin scaffolds and maturation was induced in a pulsatile bioreactor followed by seeding with endothelial cells differentiated from ASCs (63). Another study compared the proteomic profiles of normal arterial walls with tissue engineered blood vessels developed by ASCs-derived smooth muscle cells seeded into polyglycolic acid scaffolds and conditioned in a pulsatile bioreactor (62). The study identified 38 differentially expressed proteins between normal vessels and engineered grafts, the majority of which were cytoskeletal and actin-related proteins, indicating altered cytoskeletal integrity in ASCs engineered tissues (62).

The literature is rich with studies that investigate the use of endothelial progenitor cells (EPCs) as an endothelialization/cellularization source for tissue engineered vascular grafts (91–93). EPCs are progenitors that circulate in the blood and possess the ability to differentiate into mature endothelial cells and (to a lesser extent) to undergo endothelial to mesenchymal transition. There are several subtypes of cells that are classified under EPCs terminology, which are the result of the varying isolation methods modified since their first description in 1997 by Asahara et al. (94). EPCs could be isolated from different sources including umbilical cord blood, peripheral blood, and bone marrow (56). EPCs found in these sources are then cultured *in vitro* to expand, proliferate, and differentiate into endothelial cells. The main subtypes of EPCs isolated by selective culture methods include early outgrowth endothelial cells (eOECs also known as myeloid angiogenic cells; MACs, or colony forming unit endothelial cells; CFU-EC) and late outgrowth endothelial cells (also known as blood outgrowth endothelial cells; BOECs, and endothelial colony forming cells; ECFCs), and these differ in their colony formation potential, time of emergence in culture, angiogenic, and proliferative capacity and phenotypic characteristics. eOECs are CD31+, CD45+, CD14+, CD133-cells that emerge in culture between 5 and 9 days, and were described as monocytic cells that lack the ability to differentiate to endothelial cells (95). ECFCs or BOECs are produced within 7–21 days of culture on collagen coated plates (96, 97). They express the markers CD34 and CD31 and lack the expression of the hematopoietic markers CD45, CD14, and CD115 (98, 99). Other isolation methods depend on the selection of EPCs from peripheral PBMCs according to the expression of a pattern of surface antigens. These EPCs are usually isolated by positive selection of CD34+ cells, combined with other markers (such as VEGFR2 and CD133). Some studies have shown that CD34+ VEGFR2+ cells might represent cells shed from the vasculature (100). The combination of CD34+ VEGFR2+ CD133+ have resulted in contradicting findings in terms of cells ability to differentiate to endothelial cells (101). Of the identified populations, BOECs (or ECFCs) were shown to have potent proliferative capacity and can differentiate into mature endothelial cells when cultured *in vitro*. These cells also have potent angiogenic capacity and can participate in the repair of injured endothelium. Thus, BOECs (or ECFCs) are currently believed to be the “true EPCs” (92, 99). Notwithstanding the above differences, examples in the literature utilized all of the described subtypes for the development of vascular grafts. For example, Wu et al. described the isolation of CD34+/CD133+ EPCs from human umbilical cord blood, and their *ex vivo* expansion and differentiation into mature endothelial cells. The study showed that endothelial cells derived from EPCs had the ability to assemble into microvascular structures when seeded on polyglycolic acid-poly-L-lactic acid scaffolds (PGA-PLLA) with human smooth muscle cells (64). Zhou et al. reported the use of BOECs harvested from canine peripheral blood and seeded into a hybrid biodegradable polymer scaffold, which resulted in a viable vascular graft with good mechanical properties (65). Promising results were also presented using reprogrammed iPSCs, which were found to be a novel source of EPCs (66). Human iPSCs were shown to generate cells similar to BOECs (ECFEs), which are the late subset of EPCs (66). Prasain et al. demonstrated the vasculogenic characteristics and vascular repair potential of human iPSCs-derived ECFCs implanted into mice models of ischemic limbs and retinas, and the formation of human microvessels *in vivo* in immunodeficient mice (66). The isolation of other vascular cell types from the blood such as smooth muscle cells (102–105) and pericytes (106) has been described and represents a potential source for vascular graft development. For example, Aper et al. described the development of a an autologous small-caliber vascular graft using late outgrowth endothelial and late outgrowth smooth muscle cells isolated from peripheral blood progenitors and seeded on a fibrin scaffold (67).

Embryonic stem cells represent another potential source of vascular cells that can be used to develop engineered vessels. They are pluripotent stem cells that have the ability to differentiate into different cell types from the three embryonic germ layers (ectoderm, endoderm, and mesoderm), which makes them a potential candidate for tissue engineering (107, 108). The study of embryonic stem cells started with the isolation of mouse embryonic stem cells in 1981 (109, 110). It wasn't until 1998 that techniques were established to culture human embryonic stem cells (111). However, this has been associated with rigorous ethical, political and religious controversies that constrained their use up to this date (112). Methods to differentiate embryonic stem cells to vascular cells have been described (113, 114). Levenberg et al. discussed the differentiation of human embryonic derived cells into endothelial cells to form a vascular structure (115). The study showed that during certain periods of embryonic cells differentiation, an increase in endothelial cell-specific genes is detected (115). Additionally, embryonic stem cell derived vascular smooth muscle cells have been studied and utilized for blood vessel tissue engineering (113). The isolation of CD34+ vascular progenitors capable of differentiating to both endothelial and smooth muscle cells have also been described (116). Despite their differentiation potential, the use of embryonic stem cells represents a challenge due to the difficulty in cell sourcing and the ethical and regulatory concerns surrounding their use (117).

### Viability and Fitness of the Cell Source

The aim of autologous drug screening is to use patients derived cells to develop vascular grafts for personalized drug testing. The viability of the cells and their fitness to develop such systems should be taken into consideration. It is well-known that diseases such as diabetes and cardiovascular diseases reduce the viability and functionality of vascular cells and some types of stem cells such as EPCs (118, 119). While this will reflect the state of disease for each specific case, the viability of the chosen cell type is important for the development of the grafts. Reports have shown the ability to isolate and expand some of these cells to several passages from patients, while still reflecting the dysfunction related to the disease state (118). It is worth noting that extensive passaging of cells, however, might also lead to senescence and loss of phenotype and function (120, 121).

Another factor to consider is the effect of storage and cryopreservation on the isolated cells. Cryopreservation is an important step that allows the long-term storage of cells for future use. However, suboptimal cryopreservation can affect the genetic background, phenotypic stability, viability, and cell function which in turn can lead to reduced cell yield and batch to batch variability (122). Thus, cryopreservation protocols should be optimized and standardized to ensure viable, stable, and functional cells that closely reflect the phenotypes and functions of the native cells. Freshly isolated cells could be obtained from some of the previously mentioned resources (such as blood progenitor cells, iPSCs, and MSCs) but the duration of cell isolation, maturation, and differentiation (if needed) should be considered for fresh samples.

### Scaffolds

The tissue engineering technique is another key factor that should be carefully considered. Variable tissue engineering techniques have been investigated to develop vascular grafts, either supported with a scaffolding material, or composed entirely of cells (123). The main target is to provide an appropriate microenvironment for the cell source to develop a construct that mimics the native vessel. In native tissues, cells adapt to their microenvironment and change their phenotype accordingly (1). The extracellular matrix, which is the non-cellular component of the tissue's microenvironment, is a network of macromolecules that provide structural and mechanical support to the tissue (1, 124). In addition, the microenvironment of the natural tissue is a main regulator of the signaling pathways that derive cellular processes such as cell growth, differentiation and angiogenesis (1, 124–126). Thus, recapitulating the characteristics of the native microenvironment is a target for tissue engineering and 3D cell-based drug screening.

In cell-based drug screening, the main goal is to study the pharmacokinetics of drugs and their interaction with cells, and to assess any potential cytotoxicity (126). The current established methods for drug screening using 2D cultures face many limitations as they do not fully recapitulate the 3D cellular microenvironment (126). The spatial arrangement of the cells within the tissue contributes to cellular functions, differentiation, and proliferation, which is not reflected in 2D models. The cellular polarity is also different when comparing 2D to 3D models. The difference in polarity that is mediated by the arrangement of cells affects the way cells interact with their microenvironment (127). Developing better drug screening assays requires a medium that reflects the 3D cellular microenvironment that the drug will act on in the body (126). The main aim of scaffolds is to mimic the native extracellular matrix and provide structural stability to the cell culture. The introduction of scaffolds, thus, is considered to be a promising outlook for developing cheaper, quicker, and more accurate drug screening modalities (1). Scaffolds should be developed to mimic the normal scaffolding ECM, taking into consideration the mechanical characteristics, microarchitecture, and compatibility of the substrate with cell adhesion, proliferation and phenotypic expression (128).

Scaffolds can be composed of either biological materials or synthetic polymers (Table 4). The effectiveness of both modalities must be assessed as scaffolds are used in tissue engineering applications (130). Biological scaffolds could be made of decellularized tissues, small intestinal submucosa, or ECM components (131, 132). In general, decellularized tissues provide a promising template for tissue engineering because they preserve normal tissue structure and ECM content (129). However, the various processing steps these scaffolds undergo to achieve decellularization may have a negative impact on the structural integrity and mechanical properties of the ECM. This, in addition to the invasive isolation protocols needed to obtain the tissues could limit their use in drug screening applications (133). Another approach is to encapsulate cells in pure ECM hydrogels, allowing them to secrete their ECM inside the extracellular space (134). Hydrogels, which are high-water content crosslinked polymers, are commonly used in scaffolds due to their mechanical and chemical properties. Hydrogel scaffolds can be formed from natural or synthetic materials–with both having limitations in their ability to recapitulate the native ECM (127). Natural hydrogels composed of collagen, hyaluronic acid, Matrigel, chitosan, or alginate make favorable 3D cellular substrates, as they contain similar components to that of the native ECM (126). Thus, natural hydrogels are considered to be biocompatible and bioactive (127). Collagen, a natural protein in the ECM, is heavily used in tissue engineering due to its robust biocompatibility, biodegradability, and its ability to promote cell adhesion (135). One of the main disadvantages of using hydrogels in scaffolds is their limited mechanical properties (135). This has been tackled by the addition of chemical crosslinkers to improve the mechanical properties of the scaffolds.

**Table 4:** Summary of tissue engineering scaffolds types, advantages, and limitations.

**Type**	**Examples**	**Advantages**	**Disadvantages**
Biological	Decellularized tissues, small intestinal submucosa, or ECM components	• Preserve normal tissue structure and ECM content • Provide a template for cellular growth (129)	• Invasive isolation protocols • Require extensive processing for decellularization which affects the structure and mechanical properties of the ECM.
Synthetic	**Natural polymers:** • Proteins, polysaccharides, and polynucleotides	**Natural polymers:** • Contain binding sites that drive cellular processes such as differentiation and proliferation	**Natural polymers:** • Batch-to-batch differences • Less reproducible • Difficult to determine the complex interactions that occur between the scaffold and the cells (127) • Low mechanical properties
	**Synthetic polymers:** • Poly(ethylene glycol) (PEG), poly(vinyl alcohol), or poly(2-hydroxy ethyl methacrylate), and polycaprolactone	**Synthetic polymers:** • Flexible and reproducible • Longer shelf-life • High mechanical properties	**Synthetic polymers:** • Inert- lack bioactive molecules • Possible toxicity and biodegradation by-products • Material stiffness could influence cell phenotype and responses

Synthetic scaffolds can be composed of polymers from natural sources such as proteins, polysaccharides, and polynucleotides, or from synthetic sources such as poly(ethylene glycol) (PEG), poly(vinyl alcohol), polyhydroxyalkanoate, poly(2-hydroxy ethyl methacrylate), and polycaprolactone (126). In general, the main disadvantage of using natural scaffolds is that it is difficult to determine the various complex interactions that occur between the scaffold and the cells (127). Moreover, there are many batch-to-batch differences that hinder the ability to reproduce the scaffolds and maintain consistency in cellular proliferation and differentiation (127). Natural scaffolds are less reproducible when compared to synthetic scaffolds, and have low mechanical properties. Although synthetic scaffolds are not as bioactive as natural scaffolds and are considered inert, they are advantageous as they are more flexible and reproducible. Unlike natural scaffolds, synthetic scaffolds do not have binding sites that drive cellular differentiation and proliferation. To overcome this limitation, the synthetic microenvironment could be manipulated with macromolecules to allow the interaction between the scaffold and the cells (127). For example, the addition of short synthetic peptide sequences to the scaffold allows for cell-specific adhesion and influences cell-scaffold interaction (136). This enables synthetic scaffolds to mimic the ability of natural ECM to drive cellular adhesion, differentiation, proliferation, and migration. An example of this is the incorporation of arginyl-glycyl-aspartic acid peptide (RGD) into PEG scaffolds (127). RGD motif, which is a fragment of fibronectin that mediates cell binding, is used in PEG scaffolds to increase cell adhesion (127). Hybrid scaffolds composed of synthetic and biological materials has also been introduced to overcome the limitations of both types of scaffolds.

Methods to fabricate tissue engineering scaffolds include chemical vapor deposition, solvent-casting, phase separation, fused deposition modeling, electrospinning, electrospraying, jet spraying, and 3D printing. We refer the reader to a detailed review of these fabrication methods, their advantages, and limitations (137). These methods determine the structural and mechanical properties of the scaffold, thus creating a controlled microenvironment that promote cellular processes (138). This is important because a patterned microarchitecture was found to promote cell adhesion, spreading, proliferation, alignment, migration, and ECM remodeling, thus enhancing tissue formation (139–142). Additionally, the commitment of stem cells, such as MSCs, to different lineages was found to be determined by cell shape (141, 143, 144), which is influenced by the geometry and topography of the material. The fate of stem cells is also determined by the elasticity of the biomaterial, as cells will differentiate to a specific lineage when cultured on a biomimetic material of equivalent elasticity to the target tissue (145). Thus, controlling the physical cues and microarchitecture of the scaffold is an important factor for scaffold design and consequent tissue formation.

Scaffoldless tissue engineering technologies have also emerged, relying on cells' natural ability to assemble into tissues and produce ECM. This novel approach promotes the rapid development of constructs without the need for a scaffolding material (146–148). Approaches to develop scaffoldless vascular grafts include self-organization (cell sheet engineering, and bio-printing) and self-assembly techniques (149, 150). These techniques differ in their reliance on external stimuli (energy and forces) to promote tissue formation, as previously detailed by Athanasiou et al. (63). Self-organization of scaffoldless grafts is achieved in the presence of external stimuli, while self-assembly happens in absence of such stimuli, and order is achieved spontaneously.

Self-organization techniques for vascular tissue engineering include cell sheet engineering and bioprinting. Cell sheet engineering relies on the culture of monolayered cells until they reach confluence, and then the multi-layering or stacking of these layers to form the tissue. The structure is then rolled into a tubular format with the aid of a mandrel (150, 151). The earliest example of this approach used cell sheets made of smooth muscle cells or fibroblasts cultured with supplemental medium containing ascorbic acid to influence extracellular matrix formation (152). The cell sheets were then concentrically overlapped to create a tube and the cell sheets later adhered together to form a firm tissue (152). To aid in the production of these sheets, thermosensitive plates has been developed using modified polystyrene coated with a temperature-responsive polymer, which allows intact cell sheets to be lifted by decreasing the culture temperature (153, 154). This approach preserves membrane proteins, cell-cell junctions, and extracellular matrix (153, 154). Bioprinting, on the other hand, relies on the deposition of cells into a template and utilizes cells ability to secrete ECM and integrate with the provided ECM in the bioink to develop a continuous tissue with the required microstructure. This allows the precise control of the spatial arrangement and distribution of the cells and the biomaterials within the construct, thus mimicking the microstructure of their counterparts (34–38). An example of this approach is the development of anatomically correct 3D printed multi-cellular blood vessels using nanoengineered hydrogel-based cell-laden bioinks (28). Functionalization of bioprinted scaffolds holds the promise to promote cell growth, differentiation and functions and also to enhance the mechanical properties of the construct (45). Functionalization could be achieved using bioactive moieties such as growth factors/proteins, polysaccharides, oligonucleotides, and aptamers, antibodies, or short peptide ligands, and these could be incorporated in both cell-laden or cell-seeded bioprintable scaffolds using physical or chemical decoration methods (45). These molecules enhance a more biomimetic microenvironment that simulates the natural signaling and repair mechanisms thus influencing tissue formation and cellular functions (45). Bioactive inorganic fillers and nanomaterials such as graphene, graphene oxide, carbon nanotubes, calcium phosphates, bioactive glasses, silica nanoparticles, and nanoclays have also been used in hydrogel bioinks to improve printability, cell viability, and mechanical properties (155–157). These fillers could also be doped with drugs or biologically active ions to induce specific responses or act as crosslinkers (155). We refer the reader to these comprehensive reviews on the topic (155–158). As an example of functionalization, Modaresifara et al. developed a gelatin methacryloyl (GelMA) hydrogel that incorporated chitosan nanoparticles to promote growth factor delivery (158). The chitosan nanoparticles were loaded with bovine serum albumin–basic fibroblast growth factor, and their incorporation in GelMA hydrogels was shown to enhance the viability of human dermal fibroblasts (158). Schimke et al. utilized nano-scaled diamond particles that were functionalized with angiopoietin-1, and showed enhanced angiogenesis after 1 month of implantation into osseous defect in sheep calvaria (159). Such nanoparticles could be used to functionalize scaffolds to promote vessel growth (159). Gao et al. developed a vascular-tissue-specific bioink composed of vascular-tissue-derived extracellular matrix (VdECM) and alginate which allowed the formation of a biomimetic blood vessel composed of HUVECs and human aortic smooth muscle cells (160). The cell-laden bioink provided tissue specific microenvironment which enhanced cellular expression, function and tissue formation (160). Another approach is the development of self-organized organoids, an approach that utilizes organoid-forming cellular bioink for bioprinting (161, 162). Brassard et al. showed that printed intestinal and vascular constructs were geometrically guided to self-organize into lumen-containing biomimetic structures (161, 162). This approach overcomes the limitations of organoid cultures and adds more advantages to current 3D bioprinting techniques providing morphogenetic guidance and allowing more complex self-organization (74).

Self-assembly techniques rely on cells ability to secrete extracellular matrix and to develop self-organized 3D tissues derived by the differential adhesion hypothesis (146–148). In this technique, cells are seeded at a high density into a non-adherent substrate, which influences tissue assembly based only on cellular interactions, in the absence of any external forces. The cells then produce tissue specific ECM which will then mature to form the target tissue (149). The culture of cells within pre-structured substrate material guides self-assembly into highly biomimetic structures (150), in a manner that adopts the liquid-like behavior of embryonic cells (163). One example to achieve self-assembly was detailed by Gwyther et al. (148) and Strobel et al. (147). In this work smooth muscle cells were seeded into a pre-structured annular agarose well, which induced cell aggregation and self-assembly to develop tissue rings (147, 148). Strobel et al. have further described the ability to use the developed rings as building blocks to formulate tissue tubes based on ring fusion, in presence of gelatin microspheres (30) that can deliver growth factors and influence cell phenotype (31). This approach was tested using smooth muscle cells and mesenchymal stem cells. A study by Nycz et al. described an automated stacking process of smooth muscle rings onto a mandrel to develop tubular tissue engineered blood vessels (164). Similarly, Dash et al. described the development of vascular rings in agarose well systems using highly enriched functional smooth muscle cells derived from human induced pluripotent stem cells (32). Twal et al. described the use of agarose well systems in combination with cellularized microcarriers composed of gelatin microcarriers loaded with HUVECs and aortic smooth muscle cells to develop tubular structures (33). Scaffoldless tissue engineering methods may provide a quick and more convenient approach to develop vascular grafts, but this approach has some limitations. Controlling cell arrangement, cell accumulation and apoptosis/necrosis are some of the drawbacks of this approach. These drawbacks could be reduced by using technologies such as cell sheets and micropatterning.

### Structure and Function

**Figure 4:**
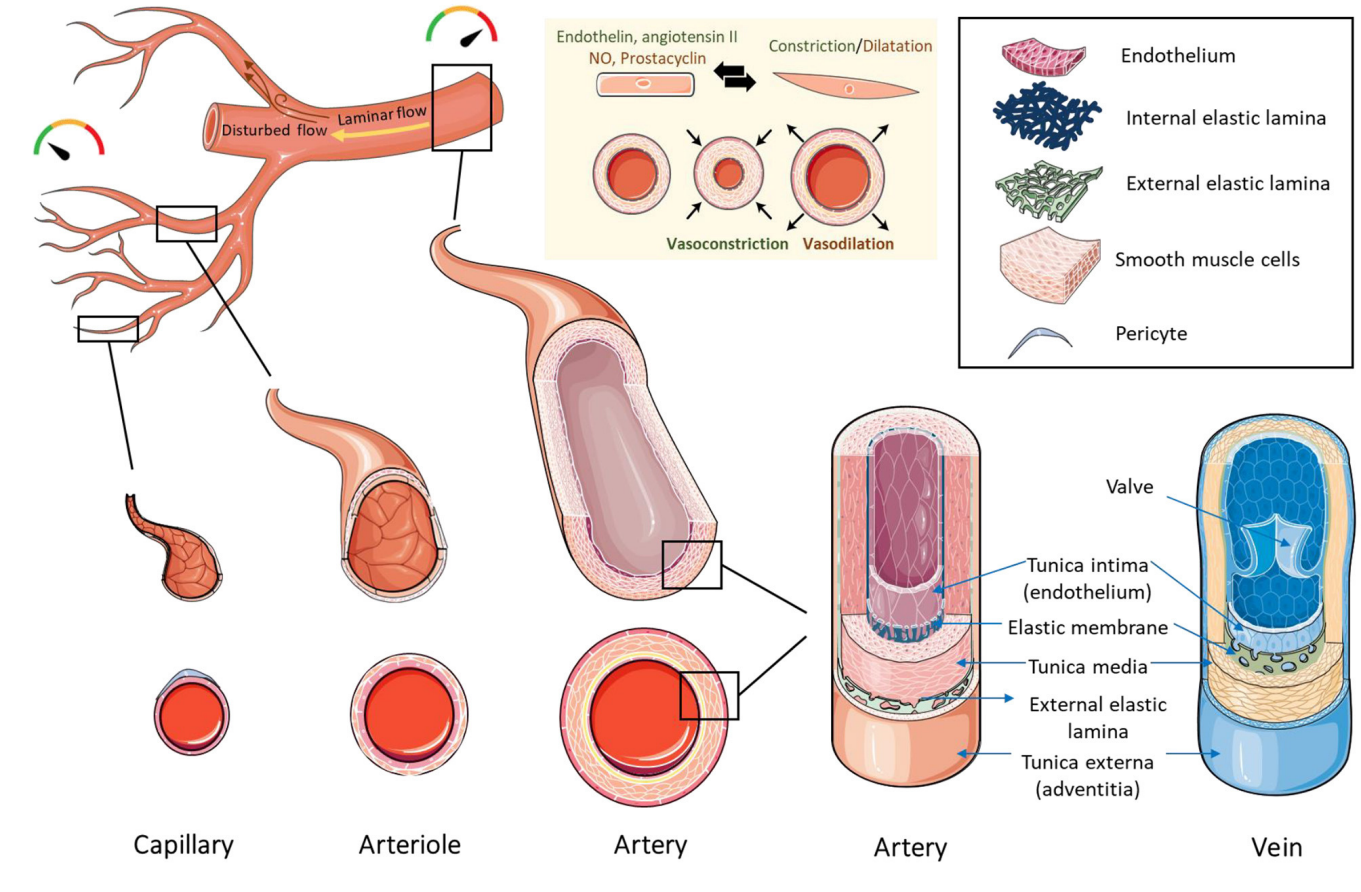
Structure of blood vessels. Capillaries have the smallest diameter and are composed of an endothelial cell layer surrounded by pericytes. Arterioles and venules are larger in caliber than capillaries and contain an endothelial layer surrounded by a few smooth muscle cells. Arteries and veins have a thick layer of smooth muscle cells and extracellular matrix in the tunica media, lined by a layer of endothelial cells in the tunica intima. Arteries and veins contain an internal elastic membrane between the tunica media and tunica intima. Arteries contain an additional external elastic lamina between the tunica media and tunica adventitia. The blood flows from the major arteries where pressure is high to small blood vessels and veins where the pressure is low. The directionality of blood flow varies according to vessel geometry. Areas of uniform geometry have unidirectional/laminar flow, while areas of branches, curves, and bifurcations, have non-directional/disturbed flow. Blood vessels control the pressure/flow of blood by changing their vascular tone. Vascular tone is maintained by the release of vasoconstrictors (such as endothelin and angiotensin II) or vasodilators (such as nitric oxide and prostacyclin) by the endothelial cell layer, which influences the constriction or dilation of smooth muscle cells, leading to vasoconstriction or vasodilation. Created using Servier Medical ART: SMART (smart.servier.com).

To achieve a reliable and accurate representation of the native blood vessel, the construct should retain the right structure and function. Native tissues are composed of cells embedded in extracellular matrix, which provides structural, mechanical, and biochemical support to the cells, and influences their behavior. Blood vessels are generally composed of endothelial cells, smooth muscle cells, and pericytes, and this composition varies depending on their location, lumen size and function (Figure 4). Arteries and veins have a tunica media composed of smooth muscle cells, collagen, proteoglycans, and elastin, which are essential for vasoconstriction and dilation. The inner part of the media is lined by an endothelial tunica intima. Smaller vessels like capillaries normally function in the exchange of nutrients and oxygen. Capillaries have a single layer of endothelium and a basement membrane, which regulates coagulation and immune cells trafficking. Arterioles and venules, which are larger in caliber to capillaries have only a few smooth muscle cells in the tunica media in addition to the endothelial tunica intima. In terms of wall thickness, arteries and arterioles have thicker walls than veins and venules due to their location in relation to the heart and their exposure to higher levels of pressure.

Physiological blood flow varies across the vasculature tree and plays a role in vascular responses and cell behavior (165). The blood flows from the major arteries where pressure is high to small blood vessels and veins where the pressure is low, and this movement results in variable forces including fluid and wall shear stresses, cyclic strains, and hydrostatic pressures (166). Additionally, the types of blood flow vary according to the geometry of the vessel. In large arteries with uniform geometry, flow and shear stress are unidirectional or laminar, while in areas of arterial branches, curves, and bifurcations, the flow is disturbed resulting in non-directional/oscillatory shear stress (167). Vascular cells respond differently to these types of flow/stresses in terms of alignment, genetic profile and secretions (168). Maintenance of a laminar shear stress is thought to be cardioprotective, through the regulation of normal physiologic vascular function, and the inhibition of proliferation, thrombosis and inflammation (169). Furthermore, it is suggested that blood flow plays an important role in vascular remodeling during embryogenesis (170). Taking cues from embryogenesis processes can guide the construction of a functional vascular tissue. Since the embryogenesis of blood vessels is out of the scope of this article, we refer the reader to these extensive reviews on the topic (171–173).

Intracellular interactions between cells of the vessel wall control functions such as vascular tone (174), and remodeling (175, 176). These interactions happen directly through gap junctions, or indirectly through paracrine signals (177, 178) and extracellular vesicles (176). Due to their position in the blood vessel wall, endothelial cells sense hemodynamic changes, biochemical signals and mechanical changes in the lumen. They then convey messages to vascular smooth muscle cells to induce vascular relaxation (such as nitric oxide and prostacyclin) or contraction (such as endothelin and angiotensin II). Vascular smooth muscle cells are characterized by phenotypic plasticity and can dedifferentiate from a contractile (differentiated) to a synthetic (dedifferentiated) phenotype, which influences remodeling and vascular tone changes (179). The contractile phenotype of smooth muscle cells facilitates vasoresponsiveness, and is characterized by low levels of proliferation, migration and extracellular matrix synthesis. Opposing to that is the synthetic phenotype, which facilitates the long-term adaptation of the vascular wall to physiological and pathological conditions through structural remodeling characterized by extracellular matrix deposition and increased cell numbers (180). Endothelial-smooth muscle cell communication also occurs in close contact sites through gap (or myoendothelial) junctions, which allows direct bidirectional exchange of molecules and ions (176). Homocellular gap junctions also exist and allow endothelial-endothelial and smooth muscle cell-smooth muscle cell communications (174). Besides these intracellular communications, interactions with circulating blood components play a role in processes such as hemostasis, inflammation, vascular repair and neoangiogenesis (181).

Another influencer of vascular cell behavior is the extracellular matrix. The extracellular matrix constitutes the major component of the vessel wall and provides physical scaffolding to the vascular cells (182). Both endothelial and smooth muscle cells secrete extracellular matrix proteins, which contribute to vessel maintenance, remodeling, and cell-matrix interactions. Structurally, collagen provides tensile strength to the vascular wall, elastin provides the elastic recoil needed to adapt to pulsatile blood flow and hemodynamic changes, proteoglycans regulate connective tissue structure and permeability and hyaluronans form a viscous hydrate gel in conjugation with water, which allows the ECM to resist compression forces (183). Furthermore, the stiffness of the extracellular matrix controls cellular behavior and processes including differentiation, remodeling, and angiogenesis (184, 185). Besides the mechanical and signaling functions of the extracellular matrix, it also acts as a template that guides cell arrangement, alignment and orientation, which also influences cellular functions.

All of these interactions play a role in physiological processes, and they also contribute to pathological processes such as atherosclerosis (176). Pathophysiological conditions affect the structures of blood vessels and alter their responses (186). Additionally, damage to the vessel wall can lead to structural and functional alterations. One example of such alterations is intimal hyperplasia, which occurs as a result of injury to the intima, characterized by increased smooth muscle cell proliferation and migration from the media to the intima and increased extracellular matrix deposition (187). A damaged endothelium leads to disruption of the vascular tone. It has also been shown that the components of the damaged blood vessel wall influence thrombosis and hypertension (188). Changes in blood pressure and mechanical stimuli also influence the structure of the vessels. Additionally, shear stress and pattern play a role in the pathogenesis of certain diseases. Areas exposed to disturbed shear stress are prone to calcification, and atherosclerotic plaques were observed to form preferentially at these locations (170). Understanding such variations in structure, function, and hemodynamic conditions are important for disease modeling and drug testing.

### Vascular Disease Relevant 3D Models

**Figure 5:**
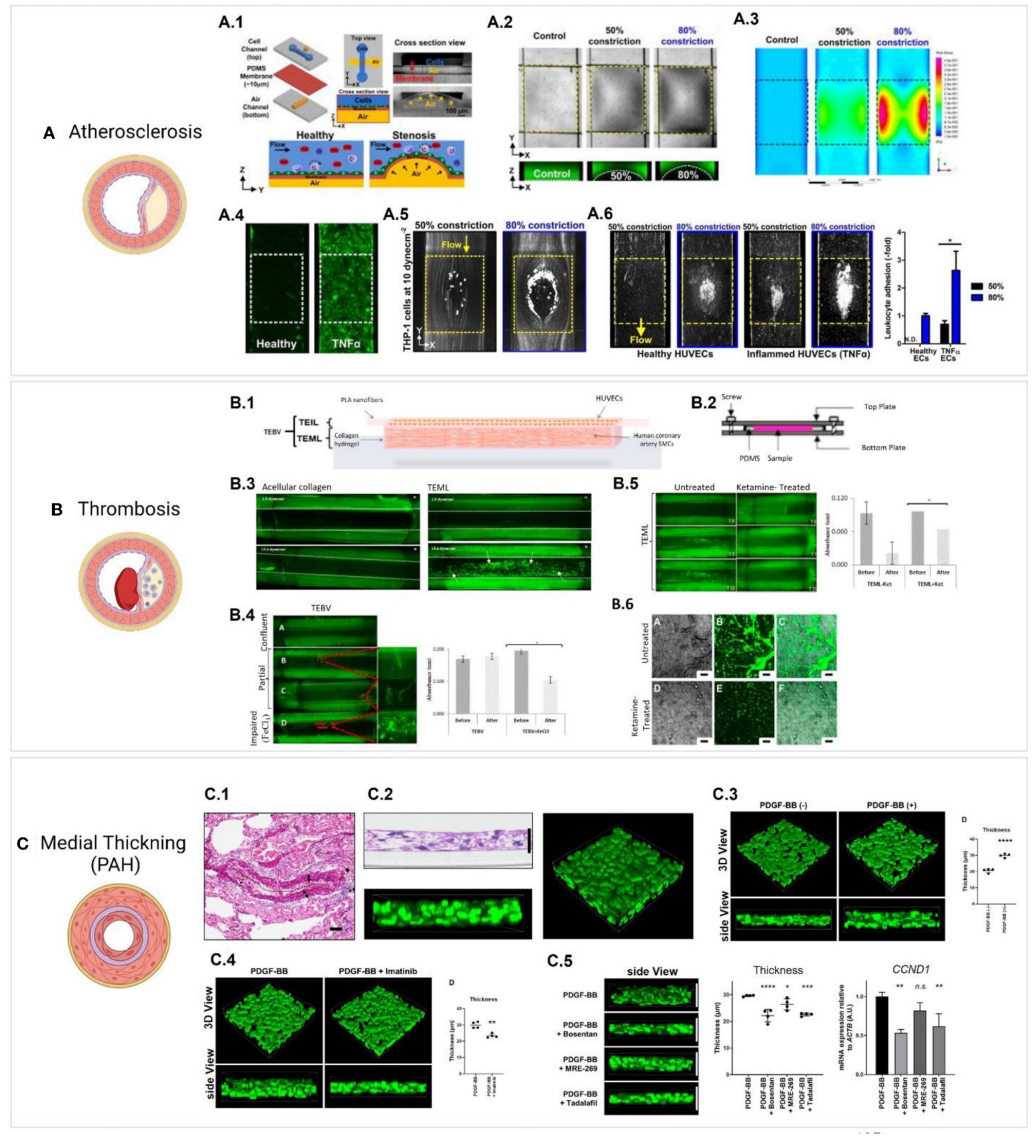
Examples of 3D vascular disease models. **(A)** Model of atherosclerosis (193). (A.1) The design of a pneumatic-controlled 3D stenosis blood vessel model composed of a cell culture channel and an air channel separated by a thin PDMS membrane (193). Pumping air into the air channel deflects the PDMS membrane upwards leading to constriction, which mimics stenotic plaque formation and vascular constriction in atherosclerosis. (A.2) Shows the channel constriction and the stenotic region (Yellow box) using bright-field images, and fluorescent images of channels loaded with FITC dye. (A.3) Fluid simulations representing wall shear stress show distinct high shear and low shear areas at 50 and 80% constriction. To study leukocyte-endothelial interactions using this model, monocytes (THP-1) were perfused over HUVECs with inflammation induced by prior treatment with TNF-α, and adhesion patterns were assessed under varying constriction degrees and shear stress conditions. (A.4) Shows the expression of ICAM1 (green) in healthy and TNF-α treated HUVECs. (A.5) shows the adherence of THP-1 to 50 and 80% constricted area at 10 dyn/cm^2^ to TNF-α treated HUVECs. (A.6) Perfusion of whole blood into the stenosis chip at 1 dyn/cm^2^ resulted in leukocyte adhesion to both healthy and TNF-α treated HUVECs following 4 h of perfusion, with inflamed HUVECs showing a significantly higher adhesion at 80% constriction. Figures were adapted from Venugopal Menon et al. (193) [Copyright 2018, licensed under a Creative Commons Attribution (CC BY) license. http://creativecommons.org/licenses/by/4.0/]. **(B)** Model of thrombosis (17). (B.1) Layer-by-layer assembly of a tissue engineered medial layer (TEML) composed of human coronary artery smooth muscle cells in type I collagen, covered with an intimal layer (TEIL) composed of a HUVEC-seeded aligned PLA nanofibers scaffold (17). (B.2) the grafts were mounted into a modified parallel-plate flow chamber, (B.3) and were then perfused with fluorescently labeled human platelets under variable flow conditions. Exposing the TEML layer (representing endothelium-denuded blood vessels) resulted in a significant platelet adhesion and aggregation. (B.4) Shows platelet aggregation and adhesion in tissue engineered blood vessels (TEBVs) with confluent, partial and impaired (treated with FeCl_3_) endothelium layer. Partial endothelium resulted in limited platelet aggregation in areas that lacked endothelial cells while impaired endothelium resulted in significant platelet aggregation on the construct. (B.5) Shows the effect of the anesthetic ketamine on platelet reactivity, which resulted in less adhesion and aggregation when compared to untreated platelets. (B.6) Shows platelet aggregates on the surface of TEML following treatment with 1 mM ketamine. Figures adapted from Njoroge et al. (17) [Copyright 2021, licensed under a Creative Commons Attribution (CC BY) license. http://creativecommons.org/licenses/by/4.0/]. **(C)** Model of medial thickening in pulmonary arterial hypertension (PAH) (23). (C.1) Reference image showing elastic tissue staining of a pulmonary artery from a PAH patient. (C.2) Generation of a 3D PAH media layer formed of human smooth muscle cells derived from PAH patients (23). (C.3) Stimulation of medial thickening was achieved using platelet-derived growth factor BB (PDGF-BB), and (C.4) the thickening is inhibited by the PDGF-BB inhibitor imatinib (1 μg/mL). (C.5) The effect of PAH drugs bosentan, MRE-269;the active metabolite of selexipag, and tadalafil was evaluated and confirmed to suppress medial thickening. Furthermore, bosentan or tadalafil reduced the mRNA expression of the proliferation marker Cyclin D1 (CCND1). Figures were adapted from Morii et al. (23) [Copyright 2020, under the terms of Creative Commons Attribution License (CC BY). https://creativecommons.org/licenses/by/4.0/].

3D drug screening systems have the potential to screen and test drugs on models that provide the disease phenotype and the correct pathophysiological settings (in terms of structure, functions, and dynamic conditions). This will provide a more accurate representation of the disease state and will provide a more reliable reflection of drugs interactions and cell/tissue responses under a more biomimetic condition. We refer the reader to a recent review on 3D models of vascular pathologies (189, 190). Systems were developed to model stenosis/atherosclerosis (191–194), intimal hyperplasia, pulmonary hypertension (23), and thrombosis (195, 196) (Figure 5). For example, Menon et al. developed a 3D stenosis blood vessel model using a microfluidic chip composed of a cell culture channel and an air channel separated by a thin PDMS membrane which is deflected upwards by air to mimic stenotic plaque formation and model vascular constriction in atherosclerosis (193). To study leukocyte-endothelial interactions using this model, monocytes (THP-1) were perfused over inflamed HUVECs (by prior treatment with tumor necrosis factor alpha, TNF-α), and adhesion patterns were assessed under varying constriction degrees and shear stress conditions (193). The utility of the system as an inflammatory profiling tool for clinical testing was further investigated by assessing leukocyte adhesion in healthy and inflamed blood (treated using different doses of TNF-α). The authors suggested the use of this device as a point-of-care blood profiling device for diabetes and dyslipidimea (193). Morii et al. have established a model for pulmonary hypertension by stimulating the thickening of a 3D media layer formed of human smooth muscle cells derived from pulmonary hypertension patients (23). Stimulation of medial thickening was achieved using platelet-derived growth factor BB, and the effect of pulmonary hypertension drugs was evaluated and confirmed to suppress medial thickening (23). Models of thrombosis have been also developed (195), including Thrombosis-on-a-Chip models (196). These models could facilitate the evaluation of novel drug candidates for these pathologies.

### Dynamic Culture Conditions

**Figure 6:**
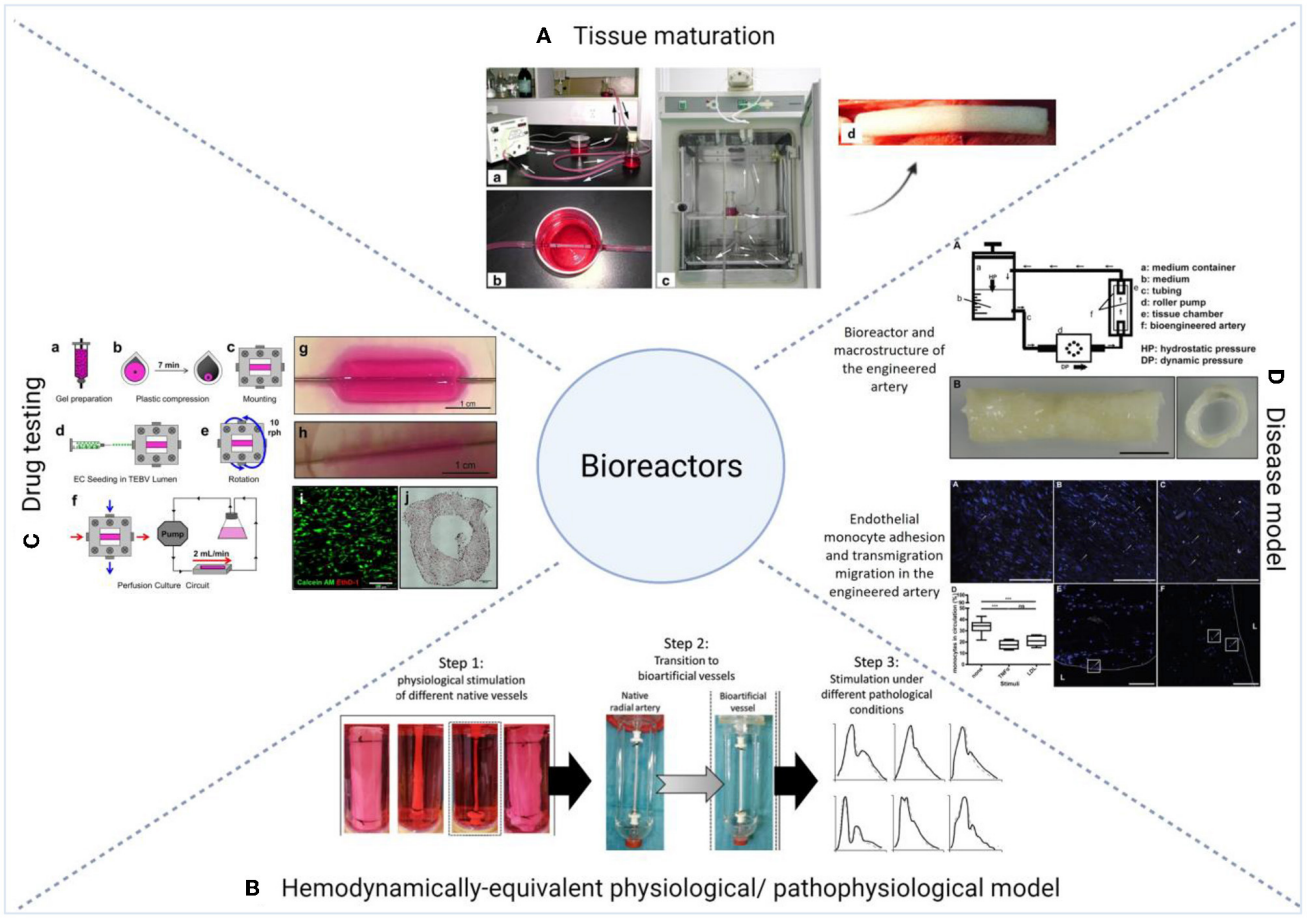
Examples of the applications of bioreactors in 3D vascular tissue engineering. **(A)** Tissue maturation. (a–d) Li et al. described the use of a custom-designed vascular bioreactor to develop small-diameter vascular grafts made of decellularized aortae of fetal pigs and canine vascular endothelial cells. Figure adapted from Li et al. (212) [Copyright licensed under Creative Commons Attribution 4.0 International License http://creativecommons.org/licenses/by/4.0/]. **(B)** hemodynamically-equivalent model. Modular hemodynamic simulator which allowed the exposure of fibrin blood vessels to site specific pressure curves, and allowed the simulation of physiological and pathological pressure conditions for small caliber vessels (213). Figure adapted from Helms et al. (213) [Copyright © 2021, The Author(s), licensed under Creative Commons Attribution 4.0 International License http://creativecommons.org/licenses/by/4.0/]. **(C)** Drug testing. Custom-made perfusion bioreactor chamber used to test pharmacological and immunological responses of tissue engineered vascular grafts made of human neonatal dermal fibroblasts or human bone marrow-derived MSCs in collagen gel (20). Figure adapted from Fernandez et al. [Copyright licensed under a Creative Commons Attribution 4.0 International License: http://creativecommons.org/licenses/by/4.0/]. **(D)** Disease model. Atherosclerosis model composed of primary HUVECs and cord blood-derived myofibroblasts cultured on a biodegradable tubular non-woven polyglycolic-acid meshes in a flow bioreactor system (191). The endothelium layer was stimulated with TNF-α or LDL and monocytes were then perfused into the system. The figure shows the adhesion of monocytes to the activated endothelium and their migration into the intima (191). Figure adapted from Robert et al. (191) [© 2013 Robert et al. licensed under the terms of the Creative Commons Attribution License]. For detailed description of the figures, readers are referred to the original articles.

Vascular cells are continuously exposed to varying hemodynamic conditions, which are also altered in various pathologies. To fully recapitulate the complex hemodynamic environment of the native vessel, flow and shear conditions should be considered. To that aim, bioreactors and microfluidic devices have been introduced (197, 198). A bioreactor is defined as “*a system that simulates physiological environments for the creation, physical conditioning, and testing of cells, tissues, precursors, support structures, and organs in vitro”* (199). Bioreactors serve two main aims: first they can be used to stimulate cell distribution, growth and expansion within the scaffolding material to influence maturation (200–205), and second they can be used to simulate physiological or pathophysiological dynamic conditions *in vitro* (206–211) (Figure 6). The types of bioreactors include static, dynamic and biomimetic bioreactors (206, 214). These systems allow for better spatial configuration and structural complexity than conventional culture methods could offer (198).

Bioreactors have been applied to create models for tissue-engineered vascular grafts, with the eventual goal of using them as *in vivo* vascular grafts (215, 216), or utilizing them to study vascular physiology and pathophysiology (191). For example, a study by Aper et al. described the use of a pulsatile bioreactor to develop an autologous small-caliber vascular graft composed of a fibrin scaffold in combination with late outgrowth endothelial and smooth muscle cells isolated from peripheral blood progenitors (67). This cultivation method resulted in a biomimetic structure and physiological biomechanical characteristics (67). Another study by Hoerstrup et al. developed pulmonary conduits by culturing human umbilical cord cells into a bioabsorbable polymer in a pulse duplicator bioreactor (217). Li et al. used a rotary bioreactor to stimulate on-site differentiation of human MSCs to vascular cells on ECM scaffolds and induce the maturation of the vascular scaffold in one system (218). As an example of a vascular disease model, Robert et al. developed an atherosclerosis system using primary HUVECs and cord blood-derived myofibroblasts which were cultured on a biodegradable tubular non-woven polyglycolic-acid meshes in a flow bioreactor system (191). To model atherosclerosis, the endothelium layer was stimulated with TNF-α or low-density lipoprotein (LDL) and monocytes were then perfused into the system. The study showed the adhesion of monocytes to the activated endothelium and their migration into the intima (191).

Bioreactors can be designed to be complementary to the type of physiology/pathophysiology desired, as vascular studies can be coupled with flow systems to study the effects on vascular cells and tissues (197, 198, 203, 219). As an example, Iwasaki et al. reported the use of a hemodynamically-equivalent pulsatile bioreactor to develop an elastic artery composed of endothelial, smooth muscle cells and fibroblasts (220). The pulsatile circulation was controlled with a left ventricular model, and the system allowed the control of dynamic flow, pressure waveforms, heart rate, and systolic fraction to match the physiological conditions of fetal or adult arteries or veins (220). Helms et al. developed a modular hemodynamic simulator which allowed the exposure of fibrin blood vessels to site specific pressure curves, and allowed the simulation of physiological and pathological pressure conditions for small caliber vessels (213).

The use of bioreactors has also been investigated for drug testing. Fernandez et al. (20) described the use of a custom-made perfusion bioreactor chamber to test pharmacological and immunological responses of tissue engineered vascular grafts made of human neonatal dermal fibroblasts or human bone marrow-derived MSCs in collagen gel. Parrish et al. (29) developed a high throughput sample-agnostic bioreactor system, that was tested on vascular grafts made of HUVECs and MSCs encapsulated in gelatin-norbornene hydrogel cast into stereolithography 3D bioprinted well inserts. The study showed the ability to induce variable flow rates in different samples of complex vascular 3D tissues. The system also allowed the cryosectioning of the grafts without removal from the insert, which increases its applicability and suitability for high-throughput mechanistic studies (29). Njoroge et al. (17) described the use of a parallel-plate flow chamber system to investigate the effect of treatments with ketamine, a common anesthetic that inhibits platelets aggregation, on EPCs recruitment using a multi-layered tissue engineered human blood vessel made of human cardiac artery smooth muscle cells and HUVECs. This demonstrated that the elimination of the anesthesia step, which is essential in animal studies, allows for a more accurate understanding of key processes such as hemostasis and vascular repair. The system was also used to model the pro- and anti-aggregatory characteristics of damaged and intact vessels under physiological flow conditions (17). These examples outline how coupling bioreactors with 3D models can better recreate the physiology/pathophysiology of the vascular tissue, which allows a better understanding of drugs effects and interactions. With the increasing complexity of such culture systems, it brings forth exciting prospects and a potential to incorporate other technologies, such as microfluidics (197).

Microfluidics represent a complex and multi-disciplinary form of technology that also employs dynamic conditions to better emulate human physiology. Microfluidics are utilized to create devices that enable the flow of fluids (in the range of micro to picolitres) in small chambers, allowing the study of fluid dynamics and its effect on adjacent cells or tissues. The use of microfluidics allows the introduction and exchange of nutrients and waste to adjacent cells or tissues, also termed lab-on-a-chip technology (219). Scientists have been able to utilize organ-on-a-chip technology with perfused microvasculature in a way that the tissue's survival is solely dependent on nutrient transport through the microvasculature within the system. Using this model, several vascularized micro-organs were plated on a 96-well plate and then used to study drug delivery to various tissue types. Furthermore, the efficacy and the toxicity of the drugs could be determined by analysis of the tissues, demonstrating the ability of such a model to be a potent means of drug screening (219).

Coupling bioreactors with microfluidic networks permits careful manipulation of fluid flow through the bioreactor system, allowing for a more physiologically relevant environment (198). Such methods allow the study of the physiology of important systems such as endothelium and vasculature, as well as other organ systems (198, 219, 221). In addition to better recreating human physiology, microfluidics has shown several other benefits when compared to traditional forms of cell culturing. It requires very small amounts of reagents, preserving valuable commodities as well as lowering waste generation. The presence of micro-chambers allows for much faster diffusion of particles, rapid heat transfer, and much faster reaction times (221). The complex 3D model can be manipulated to select specific physical parameters, such as size and charge, and chemical parameters, such as molecular composition and pharmacokinetics. This can be utilized to produce very large numbers of drug carriers with very few errors and variations (222). Moreover, complex culture models designed with bioreactors and microfluidics can be used as means of screening drugs *in vitro*, with research demonstrating it as a rapid, inexpensive, and high-throughput method, with the potential to replace the animal-testing phase in clinical studies (219, 221, 222).

The emergence of 3D printing technology has shown further promise in the field of microfluidics and bioreactors. 3D printing allows for much greater levels of customization and control over the 3-dimensional configuration of the culture system at a relatively low cost, resulting in the incorporation of finer details and hence better overall performance and resolution (198). Thus, it allows greater freedom and precision whilst fabricating bioreactors, ensuring that the cell is even more compatible and suitable for the biological system being investigated (197, 198). The greater levels of precision and manipulation, in addition to the attention to finer detail and higher performance, also ensure it to be a potent method for *in vitro* drug screening (198). An example of the application of 3D printing is its incorporation with microfluidics to develop a model that resembles the *in vivo* blood vessel networks (27). 3D printing allows for precise spatial geometry and microfluidics ensures regulated flow systems, and the combination of the two was used to create a model that greatly mimicked *in vivo* blood vessel network biology from healthy and diseased tissues (27). This model could be an effective tool to study drugs interactions with the endothelium under physiological flow conditions (27).

Another novel approach is to integrate microfluidics within the design of the scaffold to make “microfluidic scaffolds” (223–225). The purpose of these microfluidic networks is to allow the formation of a vasculature within the engineered graft to facilitate oxygen and nutrient transfer (223). This approach also allows the delivery of soluble chemicals (metabolites and signals) with temporal and spatial control (224). This will allow the study of cell responses to spatial and temporal variations of soluble factors within 3D tissues and will also prevent necrosis in thick engineered tissues (224). These microfluidic scaffolds could also be used to perfuse drugs through the built-in vasculature (225).

## Limitations, Solutions, and the Way Forward

**Figure 7:**
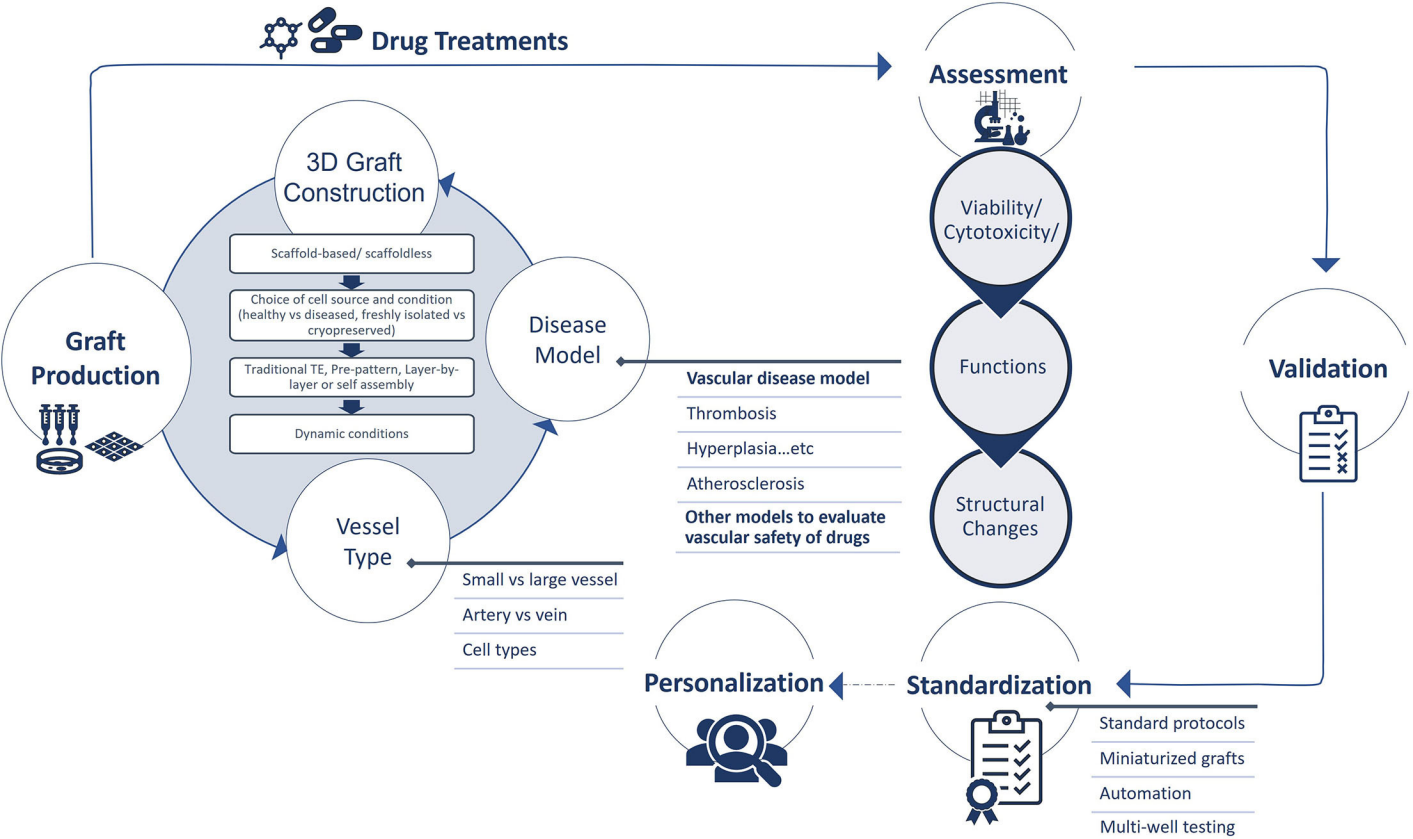
Production, assessment, validation, standardization, and personalization of 3D tissue engineered vascular grafts for drug screening.

Although the field of 3D drug screening is evolving rapidly, these models are still at their infancy, and there are many limitations that need to be overcome. The need for these systems should be evaluated to understand if they fulfill the goal of a better predictive screening for drug effects on the vascular system. This need should be balanced with relevance of these systems to the application and cost. Here we cover some of the limitations that affect the production process and assessment methods of 3D vascular drug screening systems. We also discuss a suggested pipeline to validate and standardize these systems (Figure 7).

### Production Process

No one *in vitro* biological model is perfect. It has been established by George Box in 1976 that “*Since all models are wrong, the scientist must be alert to what is importantly wrong*” (226). Awareness of the limitations of the system and any artifacts that could be created by the model design and components (such as artificial microenvironment, cell heterogeneity, intensive amounts of data, or inadequate analysis/assessment methods) are important considerations to achieve robustness. A robust system is one that can maintain functional performance despite perturbations and uncertainty (227–229). To achieve robustness and reduce variability, analysis models such as structured singular value analysis could be adopted when developing 3D drug screening systems to account for uncertainties in the design of these models (such as neglected dynamics, biological variability, dosage variations, ... etc.) (227). Uncertainties/variabilities in the 3D tissue engineering systems could arise from any of the components of the system (i.e., tissue engineering approach, cell source, “artificial” microenvironment, dynamic conditions) and this might affect the outcomes of the system and its effectiveness in predicting drug efficiency. Thus, careful selection and evaluation of the cell type, approach, biomaterial, and disease model is required to achieve robustness.

The choice of the cell types and microenvironment might affect responses to drugs. Cells are impacted by the mechanical and physical characteristics of the scaffolding material, and factors such as hydrophilicity, roughness, and stiffness should be considered when designing the system as these might affect cell responses and cell/matrix interactions (230). Furthermore, none of the investigated cell resources will exactly reflect the *in vivo* phenotypes of the cells, and this might result in varying responses. Additionally, the inherent or acquired heterogeneity of the cells in these systems could contribute to variabilities in their responses (231). Evaluation of single cell response might help in understanding whether a partial response arises from cell heterogeneity (231).

Despite the complex nature of the 3D models, they still need to be made simple enough for interpretation (227). The choice of the components of the drug screening system should be made to create a representative model (in terms of structure, composition, and pathophysiology), whilst maintaining simple and interpretable analysis methods. To reduce variabilities and create a biologically complex system, the design of the system should consider the type of the vessel (vein vs. artery, small vs. large vessel) the disease model (atherosclerosis, thrombosis, hyperplasia, deep vein thrombosis, etc.), the cell types (somatic vs. stem cells, single or co-culture models), and appropriate dynamic induction (bioreactors, microfluidics).

Besides the mentioned uncertainties resulting from the model design and components, the described 3D techniques in this review require extensive handling, high cell numbers and specialized reagents/equipment which necessitates high costs and effort. Additionally, these methods result in experimental variability caused by morphological heterogeneity, variability in cells sizes, and phenotypic instability in some of the differentiated cell types, which in all affects the structure of the final product. Tissue engineered grafts require prolonged preparation and maturation times (depending on the used cell type and tissue engineering approach). Additionally, the use of stem cells requires long complicated culture protocols and prolonged culture durations, depending on the stem cell type and the desired degree of maturation/differentiation. The best approach to overcome these limitations and to provide reliable, reproducible, and cost-effective grafts is through the development of automated miniaturized, high throughput graft production systems that will allow multi-well testing (232). Combining these miniaturized automated systems with the appropriate external stimuli (mechanical factors, growth factors, cytokines, signaling molecules) will provide a more accurate representation of the pathophysiology of the target tissue and disease state. Additionally, standardization of methods is key to reduce variability and heterogeneity between the produced vascular grafts.

### Assessment Methods

The advancements made in the field of 3D tissue engineering are not yet matched by equivalent assessment methods that can produce high throughput, high quality, and readily interpretable results. As the field of 3D culture advances, assessment methods should be modified and optimized to match these advances. Existing methods used for mammalian vascular tissues and cell monolayers should be evaluated for these systems and optimized to provide better outcomes (233). The major challenge is to create high throughput assessment methods that provide precision and repeatability, while being cost effective. The literature is relatively rich with examples of high throughput assessment methods for organoids and spheroids cultures. Thus, many of the mentioned assessment methods in this section describe these as examples that need be adjusted to accommodate the more complex 3D vascular tissues.

#### Viability/Cytotoxicity

Viability/cytotoxicity assays developed for 2D monolayer cultures should be assessed for their effectiveness in 3D cultures. The choice of the assay should be made taking into consideration the detection method, accuracy, specificity, and sensitivity (234). The interaction of the detection compound with substrates and scaffolds (such as hydrogels) should be considered. Available viability assays could either provide direct detection (by quantifying cell numbers within a specimen) or indirect detection (by assessing the metabolism or interaction of the detection compound with the cells). Various techniques are available for 2D format including colorimetric, luminometric, fluorescent, dye exclusion, and flowcytometry assays (235). Colorometric methods might not be optimal due to color absorption by some of the scaffolds' substrates. Fluorescent assays could be used, however, autofluorescence could limit the accuracy of these techniques. Flowcytometry assays will require the digestion of the 3D tissue to separate then quantify the cells. While this approach provides direct cell quantification, the processing might be challenging for high throughput applications. Additionally, digestion methods should be optimized to avoid incomplete digestion of the cells and the consequent inaccuracy in cell quantification. These methods in addition to microscopy detection approaches has been reviewed in detail by Gantenbein et al. (236).

A recent study comparing indirect viability assays in hydrogel 3D based cultures found variabilities in the output, and suggested to validate these assays with direct assessment methods (234). Other studies showed that indirect ATP- based detection assays were suitable for hydrogel-based 3D models and organoids (237, 238). Staining of cryosections using lactate dehydrogenase (LDH), calcein AM/ethidium homodimer-1, and trypan blue assay has been used and compared in chondrocytes 3D cultures (238). The comparison revealed that trypan blue was the most accurate in assessing viability/cytotoxicity. However, cryosections can only provide partial information about viability/cytotoxicity, cell distribution, and relative spatial relationship. Another study investigating the use of trypan blue to count cell numbers in spheroids digests using hemocytometer showed repeatability and reproducibility of the method, with 5% variability in estimating cell viability. However, estimating cell population density (total cell numbers per sample) showed 20% variability using the same method (239).

High throughput viability detection systems have been assessed using enzymatic conversion assays (240) and fluorescent cytometry and luminescent based assays (241). Other technologies such as the use of miniature sensors in real time to determine cell concentration in constructs has been suggested (242). Additionally, a high throughput image-based assay was described to determine the proliferation and viability of spheroids using wide field 3D fluorescence microscopy (231). In this assay, cell proliferation was determined by measuring DNA synthesis using 5-ethynyl-2′-deoxyuridine (EdU) incorporation assay, and cell viability was determined by quantifying the ratio of cells stained with a nuclear marker (Hoechst 33342) to cells labeled with cell death markers (ethidium homodimer to determine late-stage apoptosis, and extracellular apopxin to determine early-stage apoptosis). 3D image stacks were produced from each sample and analyzed layer by layer. This imaging method provides an additional advantage of assessing single cell responses to drug treatments and the discrimination between different cell populations; a useful measure for complex multi-cellular tissues (231).

The systems used to evaluate cardiovascular drugs should be integrated with other systems to evaluate drug pharmacokinetics and systematic cytotoxicity/safety. We refer the reader to a recent review on this topic (243). As an example, microfluidics has been utilized to study hepatic physiology and function, as the liver is essential for studies of drug interactions and toxicity (244). A microfluidic-based system was developed to provide human and rat hepatocyte chips a continuous flow of nutrients, which enabled the hepatocytes to be viable for over a week. The nutrients, supplied through transport vessels, were able to diffuse through the endothelial-like junctions to reach the hepatocytes, thus resembling the interaction between tissue and the vasculature. Such a physiologically relevant system enables hepatic function studies as well as drug toxicity analyses (244) in a shorter, less work-intensive, and cheaper manner than animal studies (221).

#### Vascular Tissue Functions

Functions of the vascular tissue could be evaluated by studying vasoactivity, permeability, and secretory functions. These functions could be measured using methods already optimized for vascular tissues, but with modifications (233). Vascular contraction could be evaluated using myography techniques as a measure of vasoresponsiveness, however these techniques are limited by their low throughput. Alternatively, live imaging using high resolution macroscopic imaging could be applied (245). An example is the measurement of vascular ring contraction in 96 wells plates using real-time mobile device-based imaging following the addition of drugs (246). A study by Tseng et al. utilized magnetic 3D printing; a technique by which cells are magnetized and printed using a mild magnetic force; to print vascular rings formed of smooth muscle cells, and measured vasoactive responses of these rings using real time imaging (246).

Permeability and barrier function could be evaluated using fluorescently-tagged dextran tracers. This approach was previously assessed in bioengineered microvessels (66, 247, 248), capillaries (249), vessel-on-a-chip models (250, 251), and other tubular tissue engineered structures such as intestinal models (252). Other methods such as impedance spectroscopy has been described for 3D systems (132). These approaches could be combined with studies of cell-cell junction molecules. Studying permeability under the relevant physiological or pathological dynamic conditions might provide more accurate information on vessel integrity and barrier function, as these functions are influenced by flow and shear stress (253). Additionally, external stimulus such as cytokine release under pathological/inflammatory conditions could affect permeability of the tissues and might need to be tested for each specific condition.

Tissue secretions could be evaluated by immunoassays such as enzyme-linked-immunosorbent assays (ELISA) and LUMINEX multiplex arrays or mass spectrometry analysis. These secretions could include paracrine and endocrine factors, growth factors cytokines and extracellular vesicles. 3D systems provide the advantage of multi-cellular co-cultures, which influences cell morphology and configuration and introduces cell-cell and cell-matrix interactions, thus their secretion profiles and responses differ from 2D monocultures (254–256). However, understanding the secretion profile of individual cells within the 3D tissue might represent a challenge. It is known that the hemodynamic changes modulate the secretion of inflammatory cytokines and mediators (257, 258), thus dynamic cultures should be considered when evaluating tissue secretions. Another point to consider is the artificial secretion of factors in response to the substrate or scaffold material in scaffold-based tissue engineered constructs. Additionally, the proteins supplemented in the fetal bovine serum and the growth factors in the culture media impact cell secretions, and their abundance might mask some of the secreted proteins by the cells (259).

Other functions such as leukocyte interaction and migration (193, 247, 260–262) antithrombotic properties (48, 195, 196) and angiogenesis/sprouting/neovascularization (263–266) have also been addressed in 3D models, and could be used in drug screening systems as appropriate.

#### Staining and Imaging

Conventional fixing and staining techniques used for cell monolayers will need to be optimized to ensure complete penetration and staining of the inner layers of the 3D tissues while maintaining tissue architecture and subcellular integrity. Sectioning of fixed 3D grafts followed by staining and imaging represents one approach to characterize the structure and expression of the cells. However, this approach requires relatively prolonged processing and only provides partial information about the graft structure and subcellular distribution and should be accompanied with other assessment methods. En face staining could also provide further information about the structure and expression of the grafts (267). Stereo microscopy could be used to assess structural changes and deformations in the developed grafts at a macro scale.

Imaging complete 3D grafts with small thicknesses is feasible using laser scanning confocal microscopy systems, however, imaging of thicker tissues is challenging due to light scattering (268). Recent advances has been made to reduce the scattering of light (269) and to allow imaging of thicker tissue samples (>700 μm) through multiplexed confocal imaging (270). This could be achieved by tissue clearing (or optical clearing) methods such as solvent-based clearing and aqueous-based immersion (269, 270). These techniques aim to equilibrate the refractive index of the tissues and reduce light scattering (269). These techniques could also be combined with adaptive optics to improve imaging depth and resolution (269, 271, 272).

To allow for high throughput imaging, the suggested multi-well automated systems could be accompanied with an imaging tool. Alternatively, these multiwall plate systems could be developed to be compatible with the available downstream assessment and imaging systems (273, 274). This will enhance the outcomes of 3D drug screening systems (274).

#### Data Analysis Tools

3D drug screening will result in multi-dimensional datasets that not only assess drug interactions in multi-cellular biomimetic systems, but also provide information on cell distribution, cell-cell/cell-matrix interactions, morphological effects on cell behavior, and effects of dynamic loading on cell responses to drugs within a complex setting (270). However, the use of high throughput systems combined with the complex nature of the 3D system creates massive amounts of data, and interpreting these data efficiently and correctly presents a challenge. Additionally, the heterogeneity of the tissue and cell distribution/configuration creates further challenges for the acquisition of interpretable data. Interpreting individual cell responses within the tissue when a specific cell phenotype is required will also be challenging. To design a reliable and reproducible high throughput drug screening system, suitable data analysis platforms should be implemented. We refer the reader to a recent review on the topic of data analysis in 3D drug testing (275).

### Validation and Standardization of 3D Drug Screening Systems

To provide reliable and reproducible outcomes, the 3D drug screening systems should be validated by data from human research/clinical trials (276), animal studies and 2D systems. Comparisons with data derived from healthy and diseased human tissues should be made at the biomolecular level to validate the physiological relevance of the system (268). It is important to provide an informed, unbiased and conscious evaluation of these systems to determine what they can offer to improve the existing screening strategies. The superiority of 3D systems to existing 2D systems and/or *in vivo* animal models and their ability to better predict drug responses should be confirmed before implementing these techniques for drug screening. Cost/benefit and risk/benefit analyses could help in decision making (277).

The production and assessment methods should also be standardized to reduce variability and inaccuracies in the system. This includes standardization of culture conditions, cell seeding ratios in co-culture models, and substrates/materials preparation methods (temperature, composition, arrangement/patterns, extracellular matrix components, concentrations, preparations, etc.). The standardization should also consider minimizing operation costs. The standardized models could be categorized into vascular disease models (such as thrombosis, atherosclerosis, peripheral artery disease, aneurysms, …etc.) to screen cardiovascular drugs, or non-vascular disease models (healthy vessels, or vessels stimulated to reflect the hemodynamic state of the disease or any common comorbidities) to test the safety of the drug and compatibility with the vascular system.

The advancements in the field of computational modeling could be used to optimize the design of grafts for drug screening systems (278, 279). These systems will allow the assessment of the optimal scaffold and bioreactor parameters to promote tissue formation (278, 279). They can also assist in the analysis of large-scale high content screening data sets (280, 281).

### Personalization

Is personalization the way to go? To answer this question, an understanding of personalized medicine and its applications in the context of drug testing is required. Personalized medicine definitions vary and the term is interchangeably used with other related, yet, different terms such as individualized and precision medicine (282, 283). Here, we consider the definition by the President's Council of Advisors on Science and Technology (2008) “*the tailoring of medical treatment to the specific characteristics of each patient. [It] does not literally mean the creation of drugs or medical devices that are unique to a patient. Rather, it involves the ability to classify individuals into subpopulations that are uniquely or disproportionately susceptible to a particular disease or responsive to a specific treatment”* (284). In the context of 3D drug screening, personalized medicine could be applied to test drug safety and effectiveness and optimize drug doses or combinations. Personalization could also be applied to evaluate if specific subpopulations are predicted to react differently or have adverse side effects to certain drugs based on their genetic background. Personalization in 3D drug testing could be applied by utilizing (i) autologous cells that reflect the genetic background of the subpopulation, and (ii) anatomically correct disease models that reflect the biochemical and pathophysiological profile of the disease. The use of patient-derived autologous stem cells can provide a more accurate representation of the phenotype of the disease. However, the application of personalization in 3D-based drug testing could be limited by ethical or logistic concerns related to cell sourcing and banking. The least invasive cell sources for personalization are cord and peripheral blood (blood progenitor and stem cells), skin biopsies (iPSCs) or biopsies obtained from patients during an operation (adipose derived stem cells from liposuction). Diseased tissues, cells, or blood obtained from patients could be banked for future use as a source for drug screening and disease modeling. If banking is to be considered, cryopreservation, storage and banking protocols should be optimized and standardized (285, 286). The cost of cell sourcing, expansion, and banking could be a limiting factor for these applications. Additionally, the effects of prolonged cryopreservation on cell isolation/fitness should be considered (122). The development of anatomically correct disease models is applicable (28); however, it will require high cost and specialized equipment to achieve.

We argue that personalization (rather than individualization) using 3D systems might provide a predictive, and relatively cost-effective method to screen out unsuccessful drug developments and reduce the need for clinical trials. Testing new drug developments for efficacy and safety using a representative sample of human patients is a promising approach to avoid unnecessary clinical trials (287). This approach is known as “Clinical Trials in a Dish” (287). The approach takes advantage of stem cells and their specific representation of individual patients, which allows a cohort of stem cell donors to represent a cross section of the population in a manner similar to patients recruited in a clinical trial (287). Combining this approach with the more predictive 3D models can potentially improve pre-clinical drug screening. However, this approach should be thoroughly evaluated prior to its implementation. Needless to say, standardization and validation to reduce methodological variabilities and enhance robustness are key in the success of these 3D-based Clinical Trials in a Dish (3D-CTiD).

## Conclusion

3D technologies promise to ease the economic burden of drug screening and to provide highly predictive screening systems. However, extensive evaluation and standardization of these systems is required prior to their implementation in pre-clinical drug screening. The borderline is to match the desired biological complexity of the 3D systems with throughput, reproducibility and reliability while considering the cost and effectiveness of these systems to address the drawbacks of the current 2D screening systems.

## Author Contributions

IM and CT developed the theoretical framework of the manuscript. IM, TA, MA-Q, AF and SHM wrote the first draft of the manuscript. IM, CRT, and HD reviewed and edited the manuscript. All authors read and approved the submitted version.

## Funding

This work was supported in part by a UREP award [UREP25-042-3-011] from the Qatar National Research Fund (a member of Qatar Foundation). IM was supported by the L'Oréal-UNESCO For Women in Science Young Talents Program/Middle East, 2020.

## Author Disclaimer

The statements made herein are solely the responsibility of the authors.

## Conflict of Interest

The authors declare that the research was conducted in the absence of any commercial or financial relationships that could be construed as a potential conflict of interest.

## Publisher's Note

All claims expressed in this article are solely those of the authors and do not necessarily represent those of their affiliated organizations, or those of the publisher, the editors and the reviewers. Any product that may be evaluated in this article, or claim that may be made by its manufacturer, is not guaranteed or endorsed by the publisher.
